# Three new *O*-isocrotonyl-3-hydroxybutyric acid congeners produced by a sea anemone-derived marine bacterium of the genus *Vibrio*

**DOI:** 10.3762/bjoc.16.154

**Published:** 2020-07-29

**Authors:** Dandan Li, Enjuro Harunari, Tao Zhou, Naoya Oku, Yasuhiro Igarashi

**Affiliations:** 1 Biotechnology Research Center and Department of Biotechnology, Toyama Prefectural University, 5180 Kurokawa, Imizu, Toyama 939-0398, Japan

**Keywords:** 3-hydroxybutyric acid, polyhydroxyalkanoate, sea anemone, *Tenacibaculum maritimum*, *Vibrio*

## Abstract

Liquid cultures of *Vibrio* sp. SI9, isolated from the outer tissue of the sea anemone *Radianthus crispus*, was found to produce three new *O*-isocrotonyl-3-hydroxybutyric acid derivatives, *O*-isocrotonyl-3-hydroxypentanoic acid (**1**), *O*-isocrotonyl-3-hydroxyhexanoic acid (**2**), and *O*-(*Z*)-2-hexenoyl-3-hydroxybutyric acid (**3**), together with the known *O*-isocrotonyl-3-hydroxybutyric acid (**4**). The structures of **1**–**3** were established by NMR spectroscopy and mass spectrometry, coupled with anisotropy-based chiral analysis, revealing the same *R*-configuration for all congeners **1**–**4**. The compounds **1**–**4** were weakly growth-inhibitory against a marine fish ulcer pathogenic bacterium, *Tenacibaculum maritimum* NBRC16015. Structural similarities among **1**–**4**, the *O*-isocrotonylated 3-hydroxybutyrate oligomers **5**, and microbial biopolymer polyhydroxyalkanoates (PHA) suggest the presence of a common biosynthetic machinery, and hence a possible dehydrative modification at the hydroxy terminus of PHA.

## Introduction

The genus *Vibrio*, within the class *Gammaproteobacteria*, are a group of Gram-negative, halophilic, facultatively anaerobic, rod-shaped bacteria, which are motile with sheathed polar flagella [1]. This group is one of the most widespread bacterial genera of marine origin, cataloging 128 species at the time of writing [2], of which more than 12 are known to cause enteritis, marine food poisoning, bacteremia, septicemia, cellulitis, or other infectious diseases in human and aquatic animals [3–4]. Others can fix nitrogen [5], have phototrophy [6], or produce a plant hormone [7], and thus showing a higher metabolic versatility, which is also represented by 150 and more secondary metabolites discovered from this genus [8].

**Figure 1:**
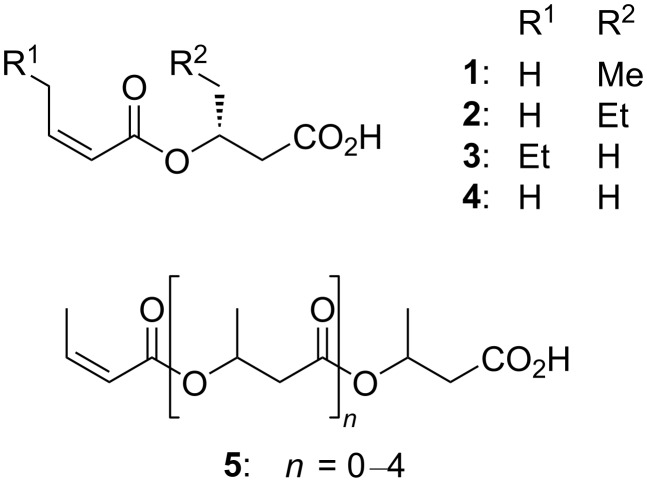
Structures of the compounds **1**–**5**.

As part of our continuing study on the secondary metabolites of marine bacteria, *Vibrio* sp. SI9, isolated from the sea anemone *Radianthus crispus*, was found to produce a known ester **4** and its new congeners **1**–**3** (Figure 1). Compound **4** is the shortest among the five oligomers of *O*-isocrotonyl-oligo(3-hydroxybutyrate) (**5**) previously discovered from *Vibrio* [9]. In this study, we describe the isolation, structure elucidation, including the absolute configuration, and bioactivity of **1**–**4**.

## Results and Discussion

The producing strain was cultured in a sea water-based medium and then extracted with *n*-BuOH. The extract was successively fractionated by silica gel chromatography using a gradient of MeOH in CHCl_3_ and octadecyldimethylsilyl (ODS) flash chromatography with elution by acidic aqueous MeCN. One of the midpolar fractions was purified by reversed-phase HPLC to give **1** (1.5 mg), **2** (11.2 mg), **3** (4.3 mg), and **4** (135.6 mg) from a 3 L culture.

The ^1^H NMR spectra of **1**–**4** similarly showed three deshielded resonances (H3, H2', and H3') and a pair of mutually coupled doublet-of-doublets resonances (H_2_2), indicating a shared core structure (Table 1, Table 2, and Supporting Information File 1). In fact, the ^13^C NMR spectra all had signals in common: two carboxy (carboxamide) and two olefinic carbon resonances along with one oxygenated carbon resonance (see Supporting Information File 1), and the analysis of an HSQC spectrum added two methyl groups and one to three aliphatic methylene groups to this composition. The molecular formula was determined to be C_9_H_14_O_4_ for **1**, C_10_H_16_O_4_ for **2** and **3**, and C_8_H_12_O_4_ for **4** by HRESIMS–TOF measurements, differing by a factor of one to two methylene units but giving the same three degrees of unsaturation, which are explained by two carboxy groups and one double bond. Thus, **1**–**4** were confirmed to be a series of acyclic compounds with a varying length of aliphatic chains.

**Table 1:** NMR data for **1** and **2** in CDCl_3_.

	**1**				**2**			
no.	^13^C	^1^H (*J* in Hz), integral	COSY	HMBC^a^	^13^C	^1^H (*J* in Hz), integral	COSY	HMBC^a^

3-hydroxy-4-methylbutyric acid					4-ethyl-3-hydroxybutyric acid			

1	175.0				176.2			
2	38.4	2.68, dd (7.3, 15.8), 1H	3	1, 3, 4	39.0	2.68, dd (7.2, 15.9), 1H	3	1, 3, 4
		2.61, dd (5.5, 15.9), 1H	3	1, 3, 4		2.60, dd (5.6, 15.8), 1H	3	1, 3, 4
3	70.8	5.22, br quint (6.3), 1H	2, 4	1, 2, 4, 5, 1'	69.5	5.25, m, 1H	2, 4	1, 2, 4, 5, 1'
4	26.9	1.71, m, 2H	3, 5	2, 3, 5	36.1	1.61, m, 2H	3, 5	2, 3, 5, 6
5	9.4	0.94, t (7.4), 3H	4	3, 4	18.4	1.35, m, 2H	4, 6	3, 4, 6
6					13.8	0.91, t (7.4), 3H	5	3, 4, 5

isocrotonic acid					isocrotonic acid			

1´	165.9				165.8			
2´	120.5	5.78, dq (11.5, 1.8), 1H	3'	1', 4'	120.5	5.74, qd (11.4, 1.8), 1H	3'	1', 4'
3´	145.6	6.34, dq (11.5, 7.3), 1H	2', 4'	1', 4'	145.5	6.30, m, 1H	2', 4'	1', 4'
4´	15.4	2.13 dd (7.3, 1.8) 3H	3'	1', 2', 3'	15.4	2.10, dd (7.3, 1.6), 3H	3'	1', 2', 3'

^a^HMBC correlations from the proton to the indicated carbon atoms.

**Table 2:** NMR data for **3** and **4** in CDCl_3_.

	**3**				**4**			
no.	^13^C	^1^H (*J* in Hz), integral	COSY	HMBC^a^	^13^C	^1^H (*J* in Hz), integral	COSY	HMBC^a^

3-hydroxybutyric acid					3-hydroxybutyric acid			

1	175.6				175.4			
2	40.5	2.73, dd (7.2, 15.9), 1H	3	1, 3, 4	40.5	2.73, dd (7.1, 15.9), 1H	3	1, 3, 4
		2.56, dd (5.7, 15.9), 1H	3	1, 3, 4		2.57, dd (5.5, 15.9), 1H	3	1, 3, 4
3	66.5	5.30, br sept (6.3), 1H	2, 4	1, 2, 4, 1'	66.5	5.32, br sept (6.3), 1H	2, 4	1, 2, 4, 1'
4	19.9	1.35, d (6.2), 3H	3	2, 3	19.9	1.35, d (6.3), 3H	3	2, 3

(*Z*)-2-hexenoic acid					isocrotonic acid			

1´	165.6				165.6			
2´	109.6	5.73, dt (11.6, 1.6), 1H	3'	1', 4'	120.5	5.76, dq (11.5, 1.6), 1H	3'	1', 4'
3´	150.9	6.23, dt (11.5, 7.5), 1H	2', 4'	1', 4', 5'	145.5	6.33, dq (11.5, 7.3), 1H	2', 4'	1', 4'
4´	31.0	2.61, qd (7.4, 1.6), 2H	3', 5'	2', 3', 5', 6'	15.4	2.13, dd (7.2, 1.9), 3H	3'	2', 3'
5´	22.3	1.46, sex (7.4), 2H	4', 6'	3', 4', 6'				
6´	13.7	0.94, t (7.3), 3H	5'	4', 5'				

^a^HMBC correlations from the proton to the indicated carbon atoms.

**Figure 2:**
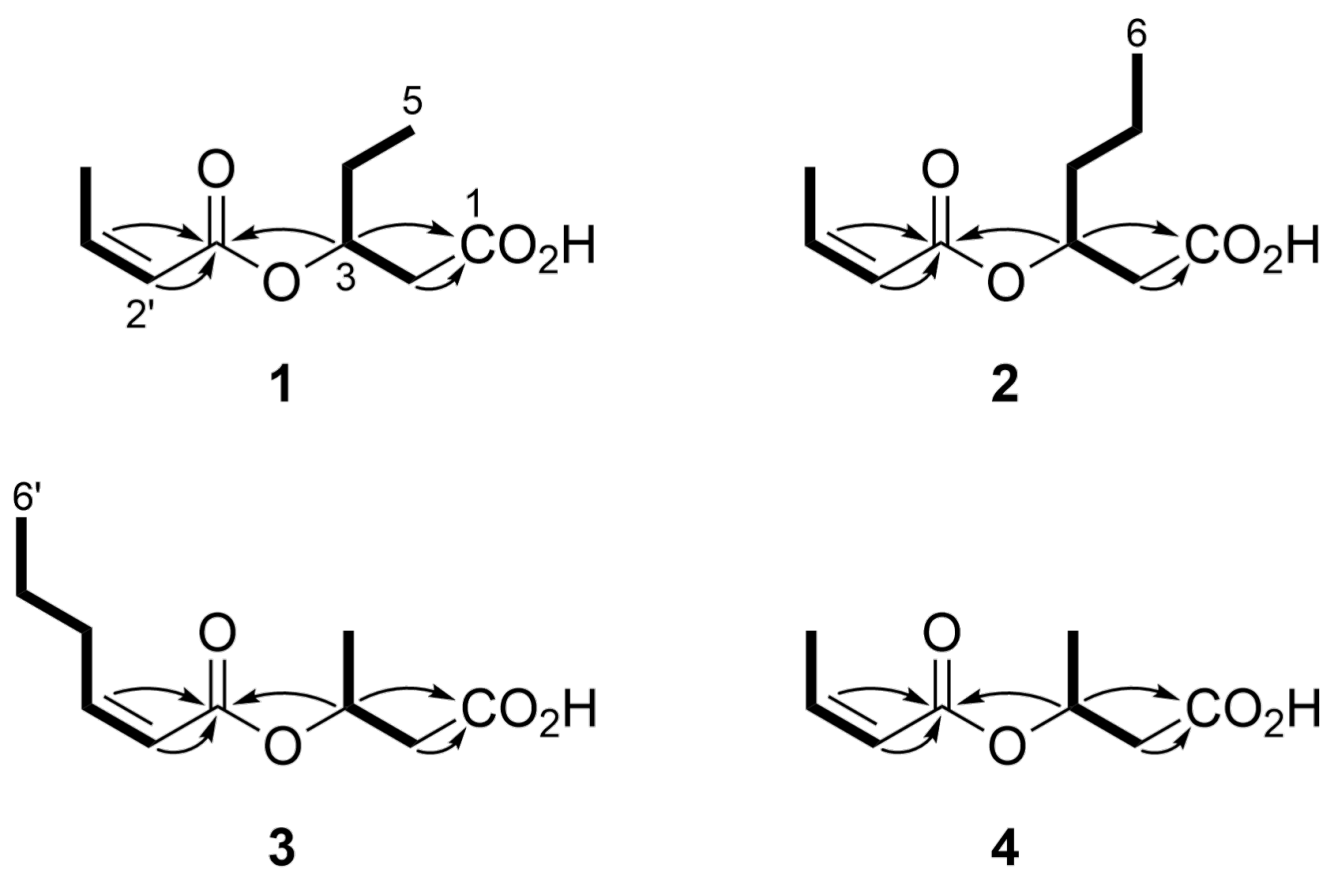
COSY (bold lines) and selected HMBC correlations (arrows) for **1**–**4**.

The analysis of a COSY spectrum of the smallest congener **4** revealed two C_3_ fragments, H_2_2–H3(O)–H_2_4 and H2'=H3'–H_3_4'. The geometry at C2' was determined to be *Z* based on a vicinal coupling constant between the olefinic protons H2' and H3' (*J* = 11.5 Hz; Table 2). Both of the fragments showed HMBC correlations to the same carboxy carbon C1 (δ_C_ 165.6), revealing an intervening ester linkage. Finally, HMBC correlations from the methylene proton H_2_2 and the oxymethine proton H3 to the other carboxy carbon C1 (δ_C_ 175.3) placed a carboxylic acid functionality on the methylene group, which completed the structure of **4** as *O*-isocrotonyl-3-hydroxybutyric acid (Figure 2).

A close similarity of the NMR data for **1**–**3** (Table 1 and Table 2) allowed the same sequence of structure analysis. The compounds **1** and **2** were found to have extra C_1_ and C_2_ extensions on the butyric acid units, while in **3**, an extra C_2_ extension occurred on the isocrotonyl group, as shown by the connectivity established by the analysis of the COSY spectra (Figure 2). Thus, **1**–**3** were concluded to be *O*-isocrotonyl-3-hydroxypentanoic acid, *O*-isocrotonyl-3-hydroxyhexanoic acid, and *O*-(*Z*)-2-hexenoyl-3-hydroxybutyric acid, respectively (Figure 1).

**Figure 3:**
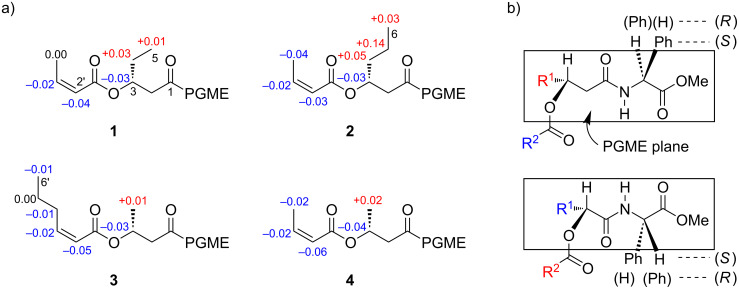
a) Distribution of positive (red) and negative (blue) Δδ_(_*_S_*_ − _*_R_*_)_ values (in ppm) calculated from the ^1^H NMR chemical shifts of the (*S*)- and (*R*)-PGME amides of **1**–**4**. b) Comparison of the major conformers of the PGME amide derivatives between the β,β-substituted carboxylic acids (top) and the α,α-substituted carboxylic acids (bottom), showing an orientational inversion of the PGME groups.

A database search identified the planar structure of **4** in a patent that described **5** (Figure 1) from marine obligate *Vibrio* sp. C-984 [9]. To determine the absolute configuration of C3 in **1**–**4**, an anisotropy-based chiral analysis using a chiral derivatization reagent, phenylglycine methyl ester (PGME), was conducted [10]. The compounds **1**–**4** were derivatized with (*S*)- or (*R*)-PGME by the action of *N*,*N´*-diisopropylcarbodiimide (DIC) and *N*,*N*-dimethylaminopyridine (DMAP) in CH_2_Cl_2_, followed by reversed-phase HPLC to give the respective (*S*)- or (*R*)-PGME amides **1a**, **1b**, **2a**, **2b**, **3a**, **3b**, **4a**, and **4b**. The calculation of the ^1^H NMR chemical shift differences Δδ_(_*_S_*_ − _*_R_*_)_ beyond C3, by subtracting the chemical shift of each proton in the (*R*)-isomer (**1b–4b**) from those in the (*S*)-isomer (**1a**–**4a**), gave positive values at C4, C5, and C6 and negative values at C2' and C3' for all four compounds (Figure 3a). Note that the sign distribution of the Δδ_(_*_S_*_ − _*_R_*_)_ values in β,β-substituted carboxylic acids is inverted from that observed in the α,α-substituted counterparts (Figure 3b) because the PGME anisotropy group is flipped upside down due to the insertion of an extra methylene group between the chiral center and the carboxylic acid functionality [11]. Thus, the *R*-configuration was concluded for all four compounds **1**–**4**.

The compounds **1**–**4** are closely related to PHAs, the energy reserve substances for eubacteria and some species of archaea [12]. Both groups of compounds are composed of (*R*)-configured 3-hydroxy fatty acids [13], and 3-hydroxybutyric acid in **3** and **4** and 3-hydroxyhexanoic acid in **2** are the most common two building blocks for PHAs [14]. However, degrees of polymerization as low as for **1**–**4** and the dehydrative modifications are unprecedented, besides for **5** [9].

Because PHAs are by nature biodegradable, can be produced from renewable bioresources, and have material properties close to the conventional petroleum-derived plastics, the commercial production and market development are actively pursued by several companies amid the growing plastic waste crisis [14–15]. *Vibrio* are perhaps the first to be known as producers of PHAs among marine microbes [16], and are isolated predominantly for the screening of the PHA production [17]. Intriguingly, aquatic farmed animals fed with poly(3-hydroxybutyrate) showed a reduced mortality compared to those not fed when being exposed to pathogenic *Vibrio* species, suggesting the application of PHAs as a biocontrol agent [18]. Although the toxicity of **1**–**4** toward the producing strain was not tested, they weakly inhibited the growth of *Tenacibaculum maritimum*, a causative agent of skin ulcers in marine fish, at MIC values of 25 (**1**), 50 (**2**), 50 (**3**), and 25 μg/mL (**4**), respectively. None of the compounds showed cytotoxicity against 3Y1 rat embryonic fibroblastic cells below 50 μg/mL.

## Conclusion

In summary, the known *O*-isocrotonyl-3-hydroxybutyric acid (**4**) and its three new congeners with different alkyl chain lengths, *O*-isocrotonyl-3-hydroxypentanoic acid (**1**), *O*-isocrotonyl-3-hydroxyhexanoic acid (**2**), and *O*-(*Z*)-2-hexenoyl-3-hydroxybutyric acid (**3**), were isolated from the fermentation extract of the sea anemone-derived bacterium of the genus *Vibrio*. The application of the anisotropy-based chiral analysis unequivocally determined the (*R*)-configuration of the 3-hydroxy acid components in **1**–**4**. These compounds showed no cytotoxicity but were weakly antibacterial against a fish ulcer pathogen, *Tenacibaculum maritimum*. The (2*Z*)-enoic acyl termini in **1**–**4** are precedented by **5**, discovered from another *Vibrio* bacterium, and the (*R*)-configured short-chain 3-hydroxy acids are the common building blocks with PHA, the microbial storage polymer. The structural similarities among **1**–**5** and PHA suggest a quite similar or even the same biosynthetic origin of these molecules, and hence a potential dehydrative modification at the hydroxy terminus of PHA. MS and NMR analyses combined with state-of-the-art chemical approaches should unveil the detailed structure of PHAs and possibly offer a clue to alter the property of these promising biomaterials.

## Experimental

### General experimental procedures

Optical rotations were recorded on a JASCO P-1030 polarimeter. UV and IR absorption spectra were recorded on a Shimadzu UV-1800 and a Perkin Elmer Spectrum 100 spectrophotometer, respectively. NMR spectra were obtained on a Bruker AVANCE 500 spectrometer, referencing to the residual solvent peaks at δ_H_ 7.26 and δ_C_ 77.0 for CDCl_3_. HRESIMS–TOF spectra were measured using a Bruker micrOTOF focus mass spectrometer. The absorbance of a formazan solution at 540 nm was measured on a ThermoFisher Scientific Multiskan Sky microplate reader.

### Biological material

The sea anemone *Radianthus crispus* was purchased from an aquarium vendor in Nagasaki, Japan. The strain SI9 was isolated from its outer tissue specimen according to the method described previously [19] and identified as a member of the genus *Vibrio* on the basis of an 98.6% similarity in the 16S rRNA gene sequence (1458 nucleotides; DDBJ accession number LC498627) to *Vibrio nereis* DSM 19584^T^ (accession number LHPJ01000025).

### Fermentation and isolation of **1**–**4**

Colonies of the strain SI9, recovered on a Marine Agar plate, were transferred into a 500 mL K-1 flask containing 100 mL 1/3 strength of simplified Marine Broth, which was prepared from 0.5% peptone, 0.1% yeast extract, and 3 L seawater, with the pH adjusted to 7.6. After being fermented at 30 °C at 200 rpm for 2 days, 3 mL aliquots of the seed culture thus prepared were dispensed into 500 mL K-1 flasks, each containing 100 mL A16 production medium consisting of 2% glucose, 1% Pharmamedia, 0.5% CaCO_3_ and 1% Diaion HP-20 in natural seawater. After being shake-cultured at 30 °C at 200 rpm for 5 days, each production culture received 100 mL *n*-butanol, and the flasks were shaken for an additional 1 h for extraction. The emulsified broth was centrifuged at 6000 rpm for 10 min, and the resulting butanol layers were collected and concentrated in vacuo to give 5.8 g of an extract from a 3 L culture. This was first fractionated on a silica gel column with a stepwise elution by CHCl_3_/MeOH mixtures (1:0, 20:1, 10:1, 4:1, 2:1, 1:1, and 0:1, v/v). The third fraction (1.35 g) was further fractionated by ODS column chromatography, eluting with MeCN/0.1% HCOOH (2:8, 3:7, 4:6, 5:5, 6:4, 7:3, and 8:2, v/v). The fourth fraction (0.28 g) was subjected to reversed-phase HPLC on a Cosmosil AR-II column (10 × 250 mm), eluting with 32% MeCN in 0.1% HCOOH at 4 mL/min to give **1** (1.5 mg; *t*_R_ 16.1 min), **2** (11.2 mg; *t*_R_ 17.6 min), **3** (4.3 mg; *t*_R_ 18.3 min), and **4** (135.6 mg; *t*_R_ 9.8 min).

### Preparation of the (*S*)- and (*R*)-phenylglycine methyl ester amides

To a solution of **4** (1.7 mg, 10 μmol) in anhydrous CH_2_Cl_2_ (0.5 mL) in a dried vial were added (*S*)-phenylglycine methyl ester (PGME, 3.3 mg, 16.4 μmol), DMAP (1.2 mg, 10 μM), and DIC (3.0 μL, 19.5 μmol), and the mixture was stirred for 30 min at room temperature. The production of the amide was monitored by thin-layer chromatography (TLC), developed using ethyl acetate/*n*-hexane 1:1, followed by heating with phosphomolybdic acid. After removing the solvent, the residue was subjected to HPLC on a Cosmosil π-NAP column (10 × 250 mm), eluted by a gradient method (MeCN/0.1% HCOOH) at 4 mL/min, with monitoring at 254 nm, to give the (*S*)-PGME amide **4a** (2.9 mg, 93%) as colorless oil.

The PGME amides **1a**/**b**–**3a**/**b** and **4b** were prepared by the same procedure but replacing the starting material and the chiral reagent accordingly.

### Antibacterial assay

The antibacterial activity was evaluated by a microculture technique described previously [20], except for a 1:100 reduction of the seeding density of *T. maritimum* NBRC16015.

### Cytotoxicity assay

3Y1 rat embryonic fibroblastic cells were maintained in low-glucose DMEM medium containing ʟ-glutamine and phenol red (Fujifilm Wako Pure Chemical, 041-29775), supplemented with 10% fatal bovine serum, 100 units/mL penicillin, 100 μg/mL streptomycin, 0.25 μg/mL amphotericin B (Fujifilm Wako Pure Chemical, 161-23181), and 100 μg/mL gentamicin sulfate (Fujifilm Wako Pure Chemical, 078-06061). The cells were seeded in each well of a 96-well culture plate at a density of 2500 cells/well. Meanwhile, the compounds **1**–**4** and doxorubicin hydrochloride as a positive control were serially diluted 1:3.16 (half-log dilution) by the same medium in a different microtiter plate. After incubating the cell culture for 12 h at 37 °C in an atmosphere of 95% air and 5% CO_2_ saturated with H_2_O, the drug solutions were transferred into each cell culture to make up 200 μL of the culture. The highest concentration of the vehicle solvents (MeOH or DMSO) was set at 0.5 vol %, where the growth of the cells was not affected. After incubating the test plate for 84 h, 100 μL of the medium containing MTT 1 mg/mL was added to each well, and the plate was further incubated for 4 h. The medium was carefully removed by aspiration, and formazan dye formed at the bottom of the wells was solubilized by the addition of 150 μL DMSO. The respiration of live cells was quantified by the measurement of the absorption at 540 nm by a microplate reader. The experiment was run in a triplicate, and the rates of the cell-growth inhibition at each concentration were plotted on a single logarithmic chart to deduce the GI_50_ values of the test compounds.

## Supporting Information

File 1 Spectra and compound characterization data for **1**–**4** and the PGME amide pairs **1a**/**b**–**4a**/**b**.
