# Supplementary material for: Three new O-isocrotonyl-3-hydroxybutyric acid congeners produced by a sea anemone-derived marine bacterium of the genus Vibrio
Source: Beilstein J Org Chem. 2020 Jul 29;16:1869–74. doi: 10.3762/bjoc.16.154 (PMC7404252; doi:10.3762/bjoc.16.154)
Supplement: File 1 — Spectra and compound characterization data for 1–4 and the PGME amide pairs 1a/b–4a/b. [file Beilstein_J_Org_Chem-16-1869-s001.pdf]

BEILSTEIN JOURNAL OF ORGANIC CHEMISTRY

Supporting Information

for

Three new *O*-isocrotonyl-3-hydroxybutyric acid congeners produced by a sea anemone-derived marine bacterium of the genus *Vibrio*

Dandan Li, Enjuro Harunari, Tao Zhou, Naoya Oku and Yasuhiro Igarashi

Beilstein J. Org. Chem. **2020,** *16,* 1869–1874. doi:10.3762/bjoc.16.154

Spectra and compound characterization data for 1–4 and the PGME amide pairs 1a/b–4a/b

License and Terms: This is a supporting information file under the terms of the Creative Commons Attribution License (http://creativecommons.org/licenses/by/4.0). Please note that the reuse, redistribution and reproduction in particular requires that the authors and source are credited.
The license is subject to the *Beilstein Journal of Organic Chemistry* terms and conditions: (https://www.beilstein-journals.org/bjoc)

Table of contents

(*R*)-*O*-Isocrotonyl-3-hydroxypentanoic acid (**1**)

UV spectrum....S2
IR spectrum....S3
^{1}H NMR spectrum ($CDCl_3$, 500 MHz) S4
^{13}C NMR spectrum (($CDCl_3$, 125 MHz) S5
COSY spectrum ($CDCl_3$, 500 MHz) S6
HSQC spectrum ($CDCl_3$, 500 MHz) S7
HMBC spectrum ($CDCl_3$, 500 MHz) S8
^{1}H NMR spectrum of (*S*)-PGME amide (**1a**) ($CDCl_3$, 500 MHz) S9
^{1}H NMR spectrum of (*R*)-PGME amide (**1b**) ($CDCl_3$, 500 MHz) S10

(*R*)-*O*-Isocrotonyl-3-hydroxyhexanoic acid (**2**)

UV spectrum....S11
IR spectrum....S12
^{1}H NMR spectrum ($CDCl_3$, 500 MHz) S13
^{13}C NMR spectrum ($CDCl_3$, 125 MHz) S14
COSY spectrum ($CDCl_3$, 500 MHz) S15
HSQC spectrum ($CDCl_3$, 500 MHz) S16
HMBC spectrum ($CDCl_3$, 500 MHz) S17
^{1}H NMR spectrum of (*S*)-PGME amide (**2a**) ($CDCl_3$, 500 MHz) S18
^{1}H NMR spectrum of (*R*)-PGME amide (**2b**) ($CDCl_3$, 500 MHz) S19

(*R*)-*O*-(*Z*)-2-hexenoyl-3-hydroxybutyric acid (**3**)

UV spectrum....S20
IR spectrum....S21
^{1}H NMR spectrum ($CDCl_3$, 500 MHz) S22
^{13}C NMR spectrum ($CDCl_3$, 125 MHz) S23
COSY spectrum ($CDCl_3$, 500 MHz) S24
HSQC spectrum ($CDCl_3$, 500 MHz) S25
HMBC spectrum ($CDCl_3$, 500 MHz) S26
^{1}H NMR spectrum of (*S*)-PGME amide (**3a**) ($CDCl_3$, 500 MHz) S27
^{1}H NMR spectrum of (*R*)-PGME amide (**3b**) ($CDCl_3$, 500 MHz) S28

(*R*)-*O*-Isocrotonyl-3-hydroxybutyric acid (**4**)

UV spectrum....S29
IR spectrum....S30
^{1}H NMR spectrum ($CDCl_3$, 500 MHz) S31
^{13}C NMR spectrum ($CDCl_3$, 125 MHz) S32
COSY spectrum ($CDCl_3$, 500 MHz) S33
HSQC spectrum ($CDCl_3$, 500 MHz) S34
HMBC spectrum ($CDCl_3$, 500 MHz) S35
^{1}H NMR spectrum of (*S*)-PGME amide (**4a**) ($CDCl_3$, 500 MHz) S36
^{1}H NMR spectrum of (*R*)-PGME amide (**4b**) ($CDCl_3$, 500 MHz) S37

Compound characterization data....S38

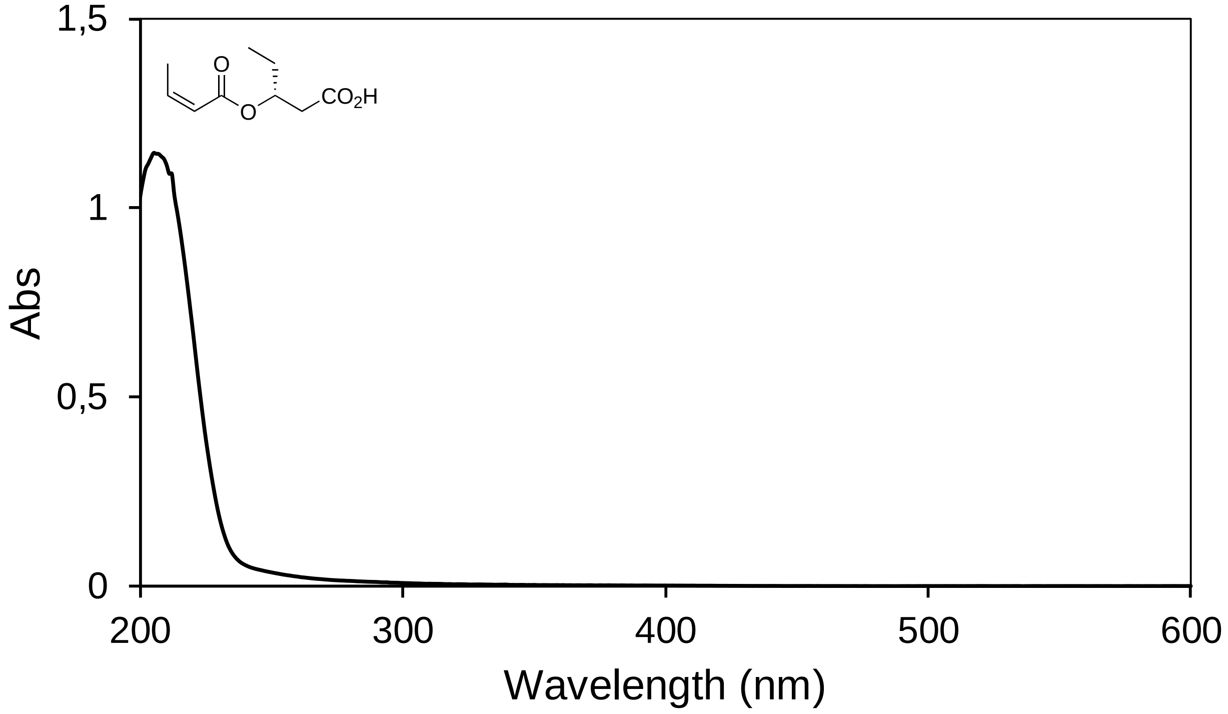

UV spectrum of **1**

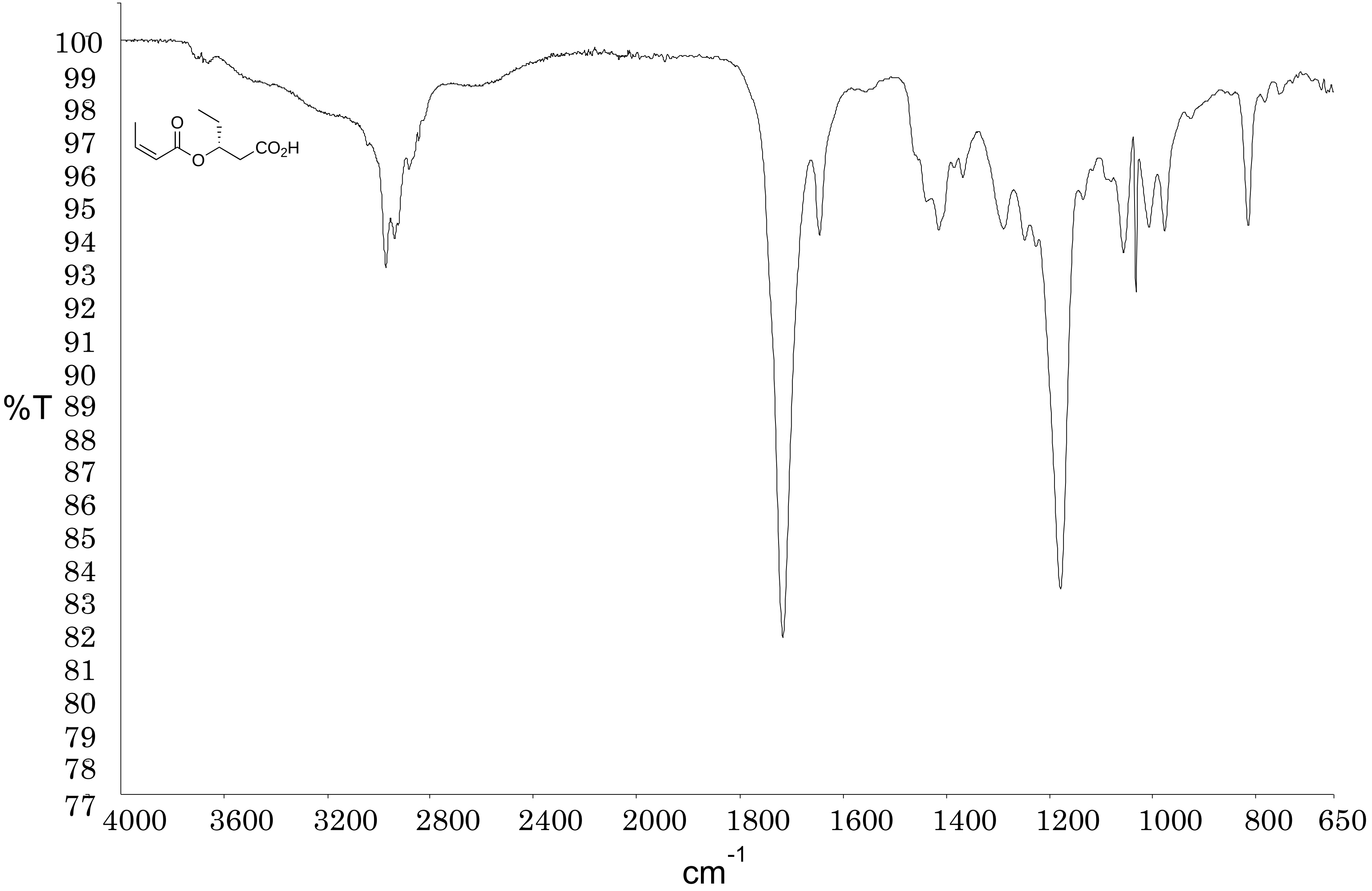

IR spectrum of **1**

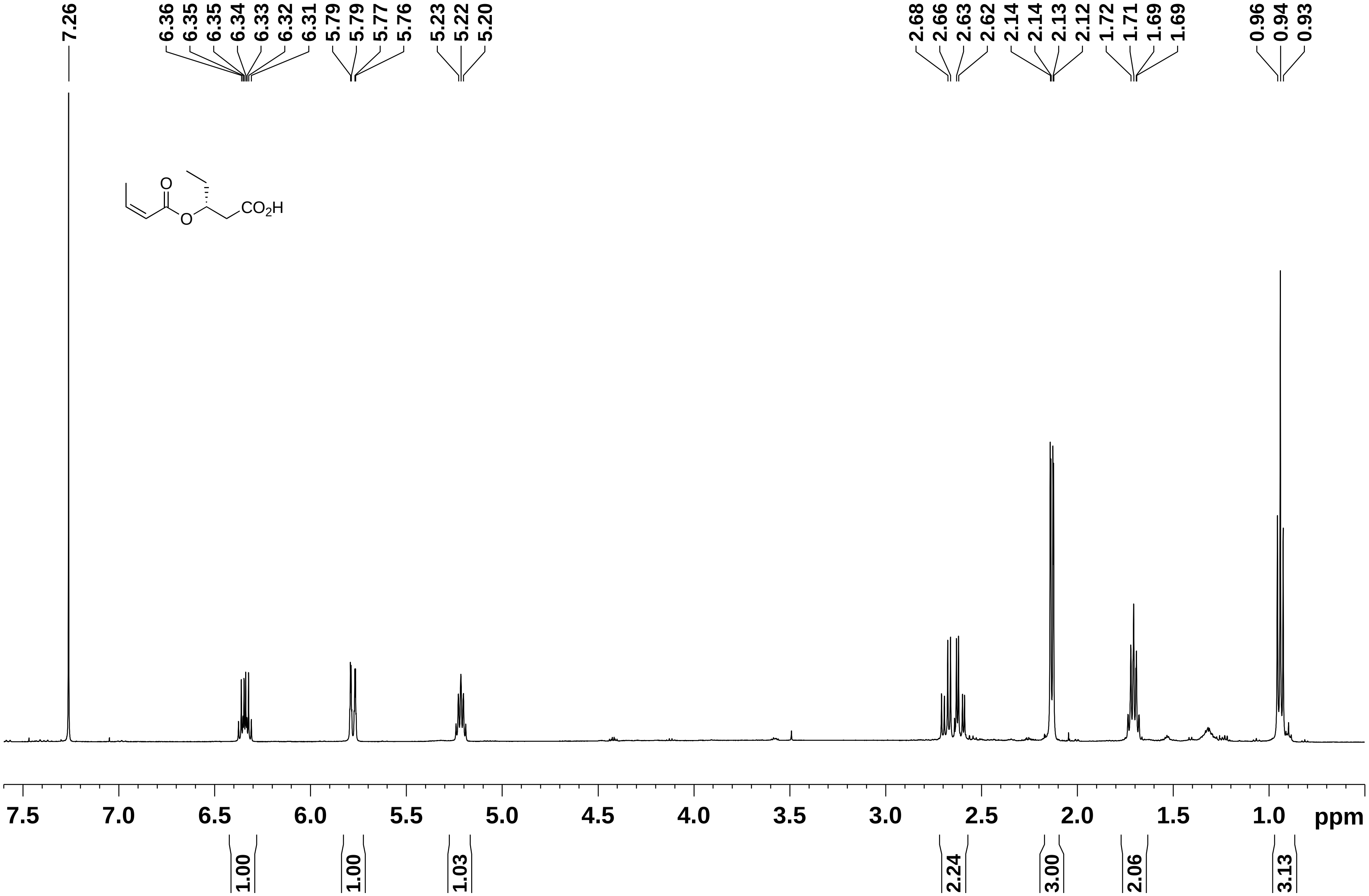

^{1}H NMR spectrum of **1** ($CDCl_3$, 500 MHz)

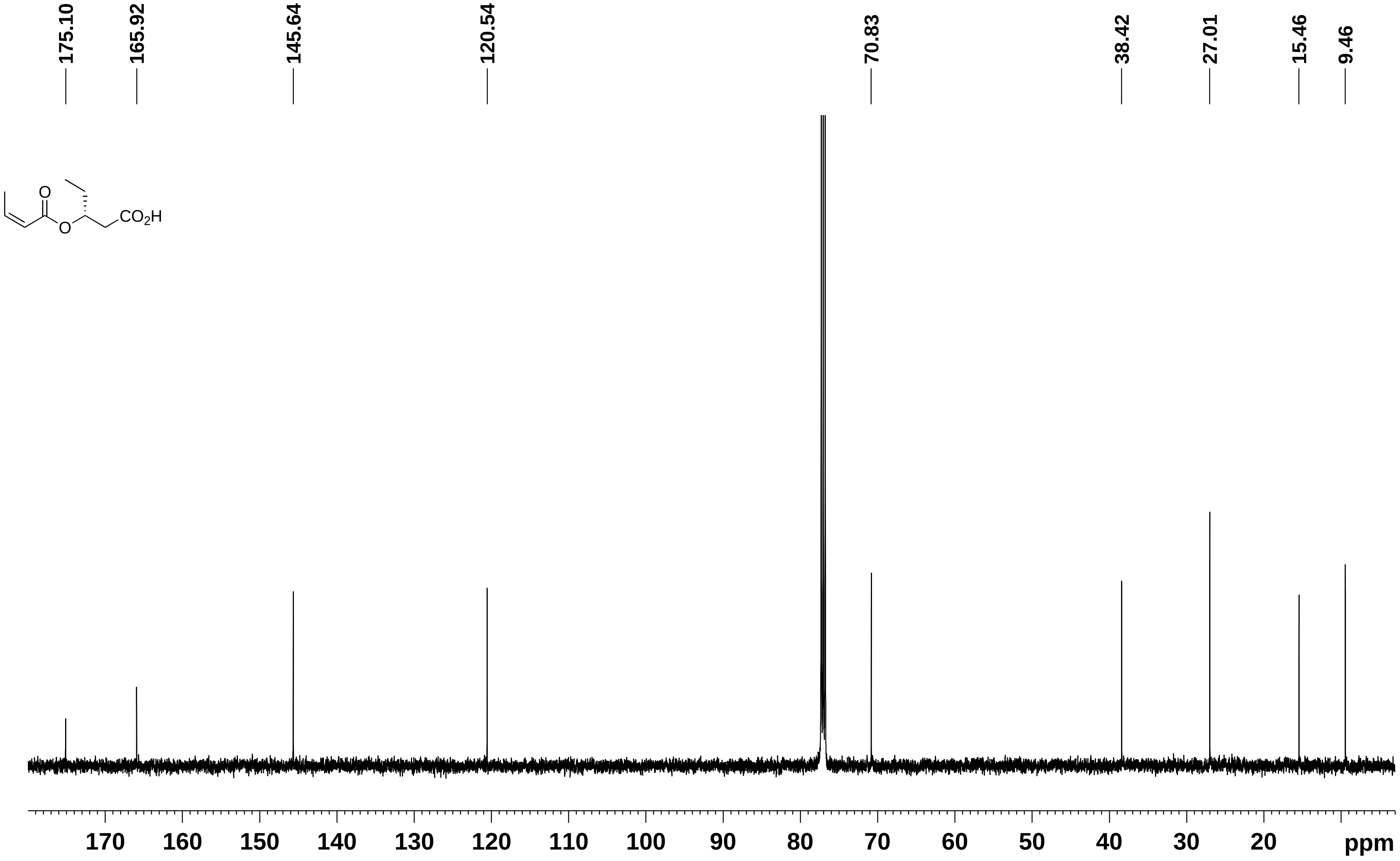

^{13}C NMR spectrum of **1** ($CDCl_3$, 125 MHz).

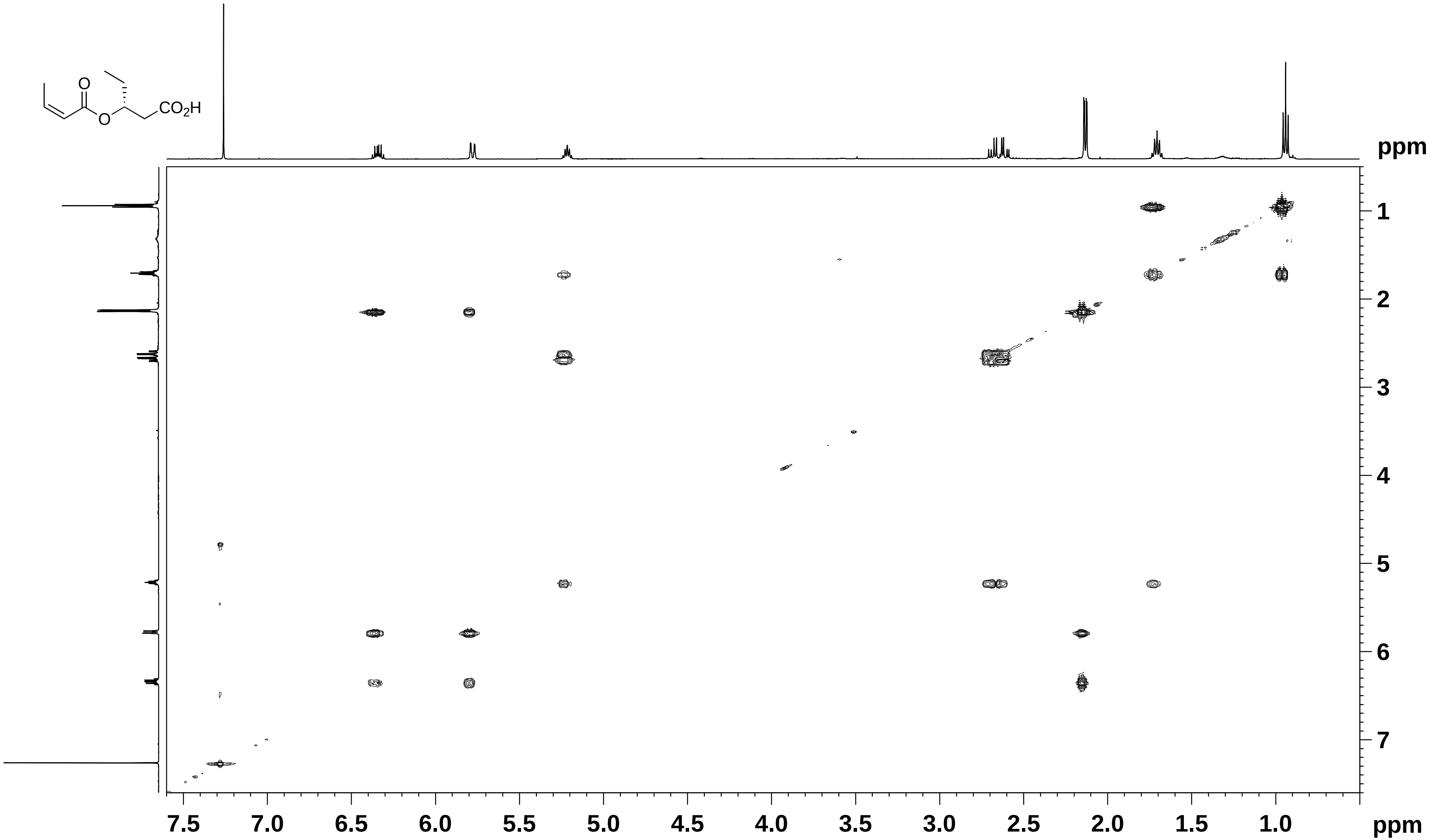

COSY spectrum of **1** ($CDCl_3$, 500 MHz).

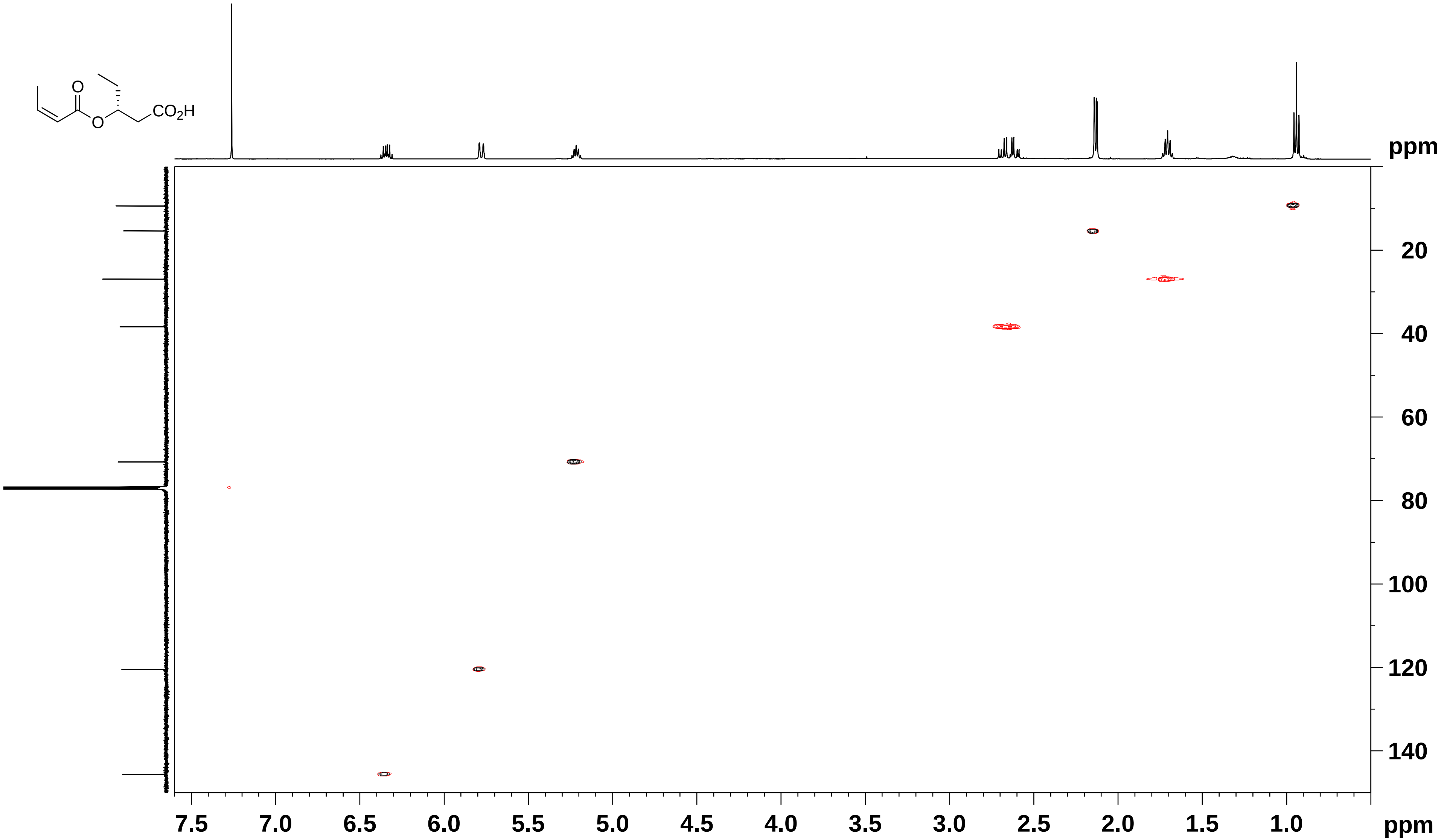

HSQC spectrum of **1** ($CDCl_3$, 500 MHz).

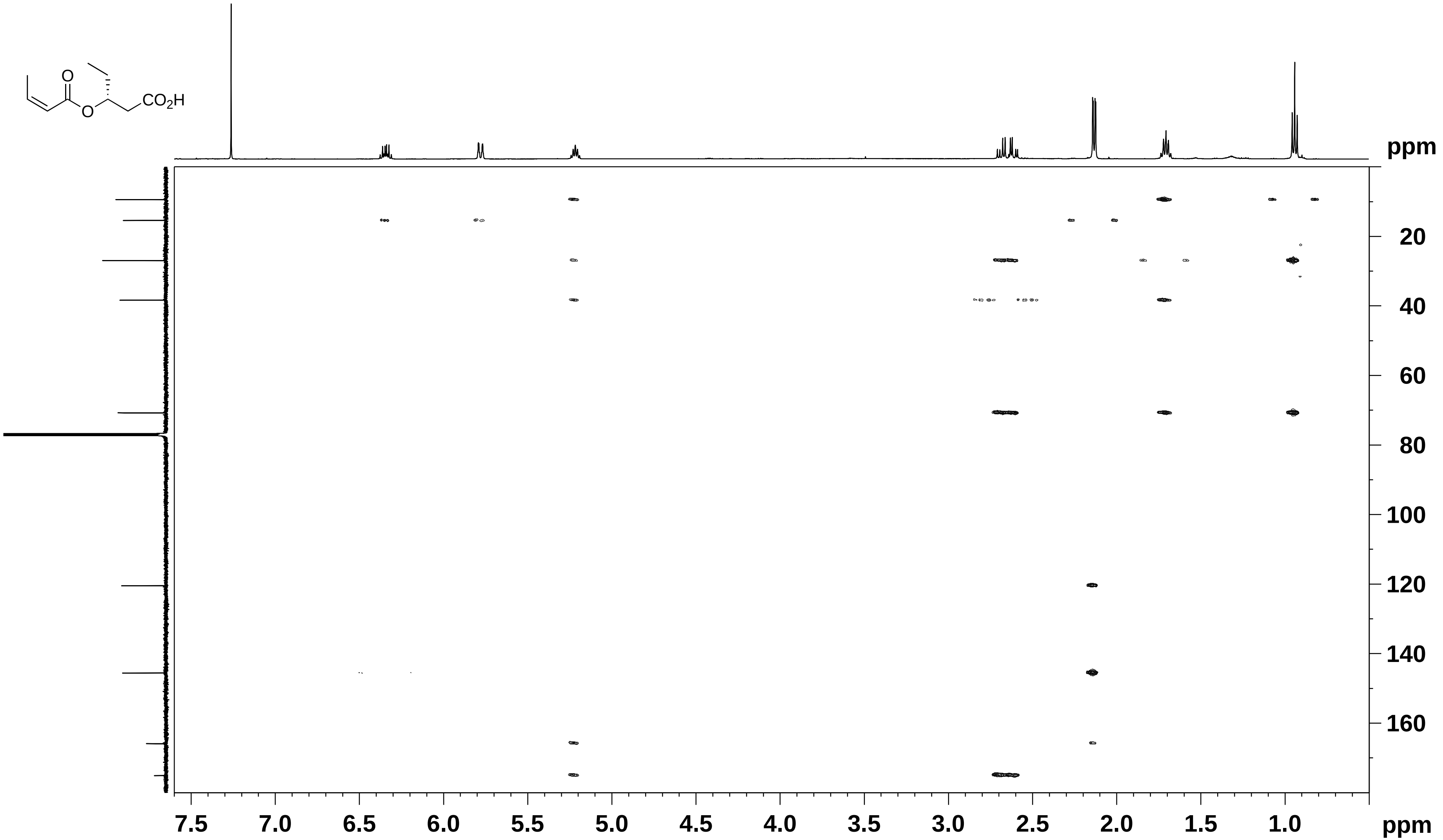

HMBC spectrum of **1** ($CDCl_3$, 500 MHz).

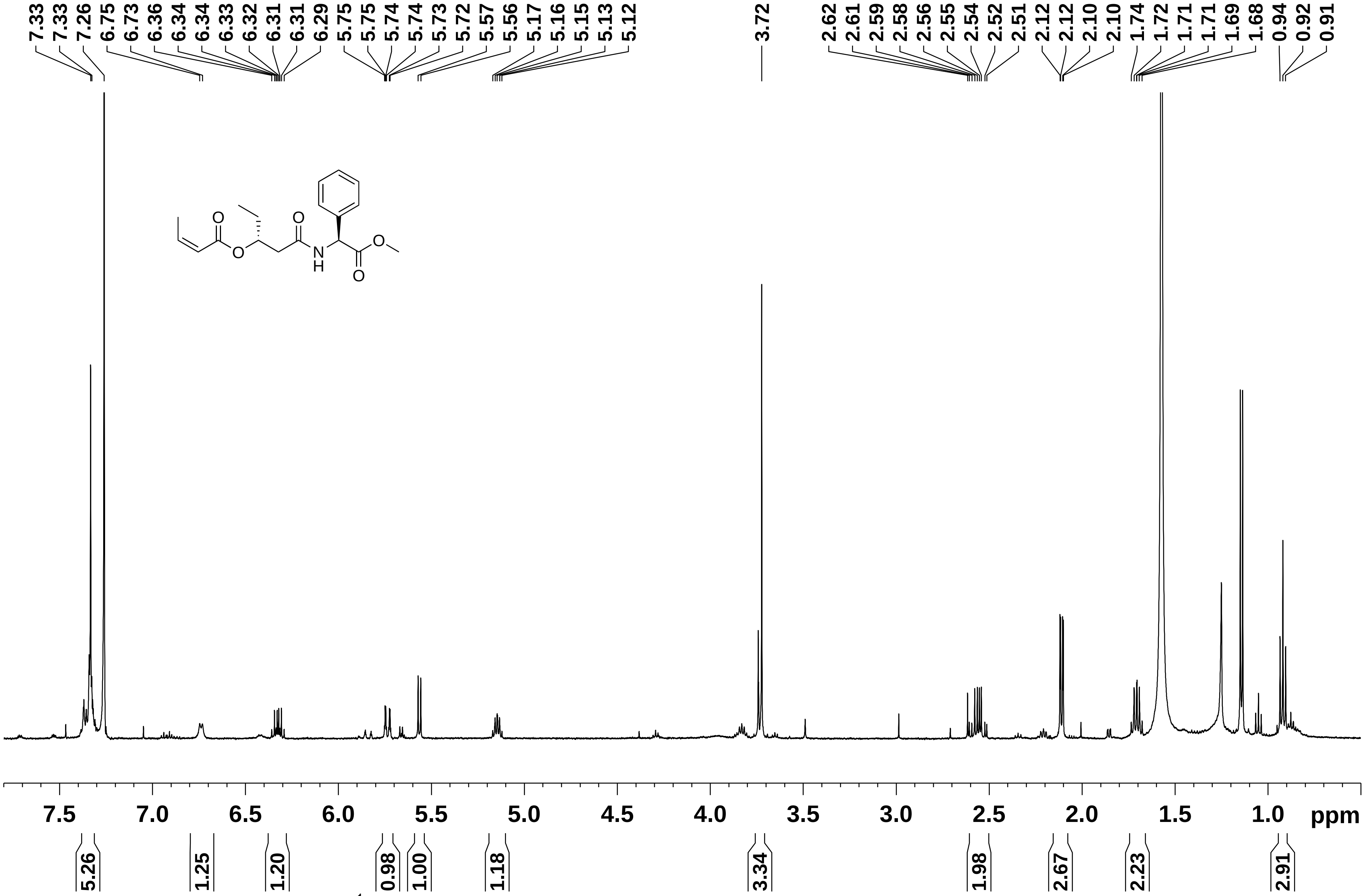

^{1}H NMR spectrum of (*S*)-PGME amide (**1a**) ($CDCl_3$, 500 MHz)

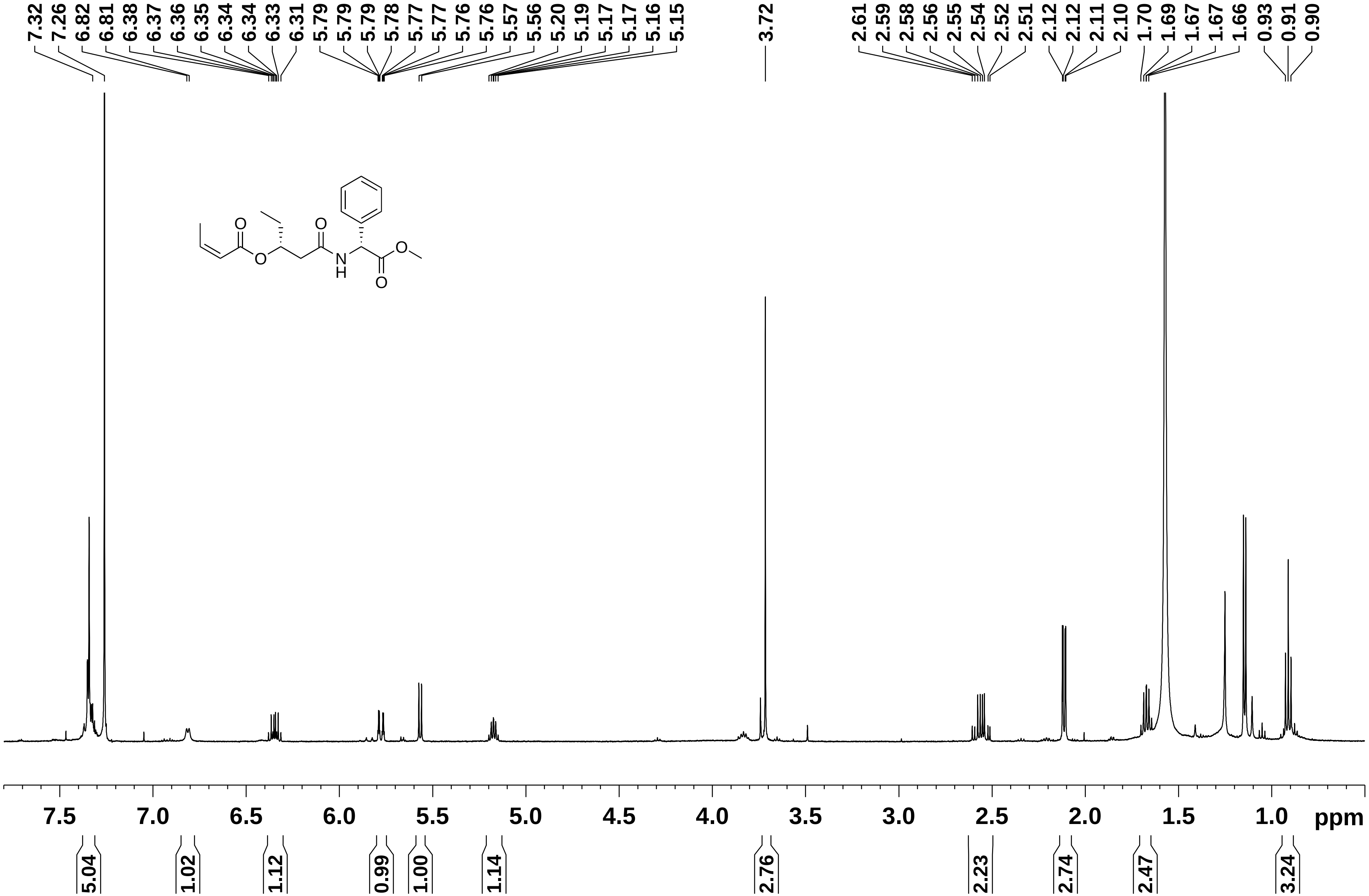

^{1}H NMR spectrum of (*R*)-PGME amide (**1b**) ($CDCl_3$, 500 MHz)

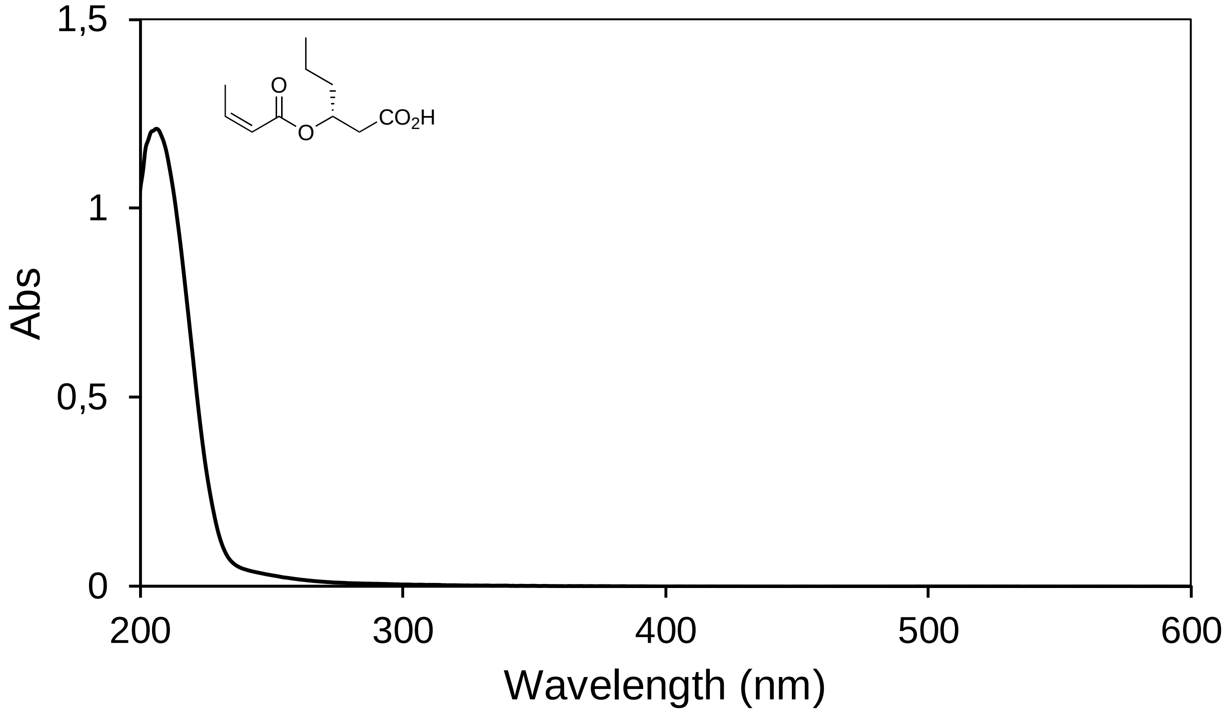

UV spectrum of **2**

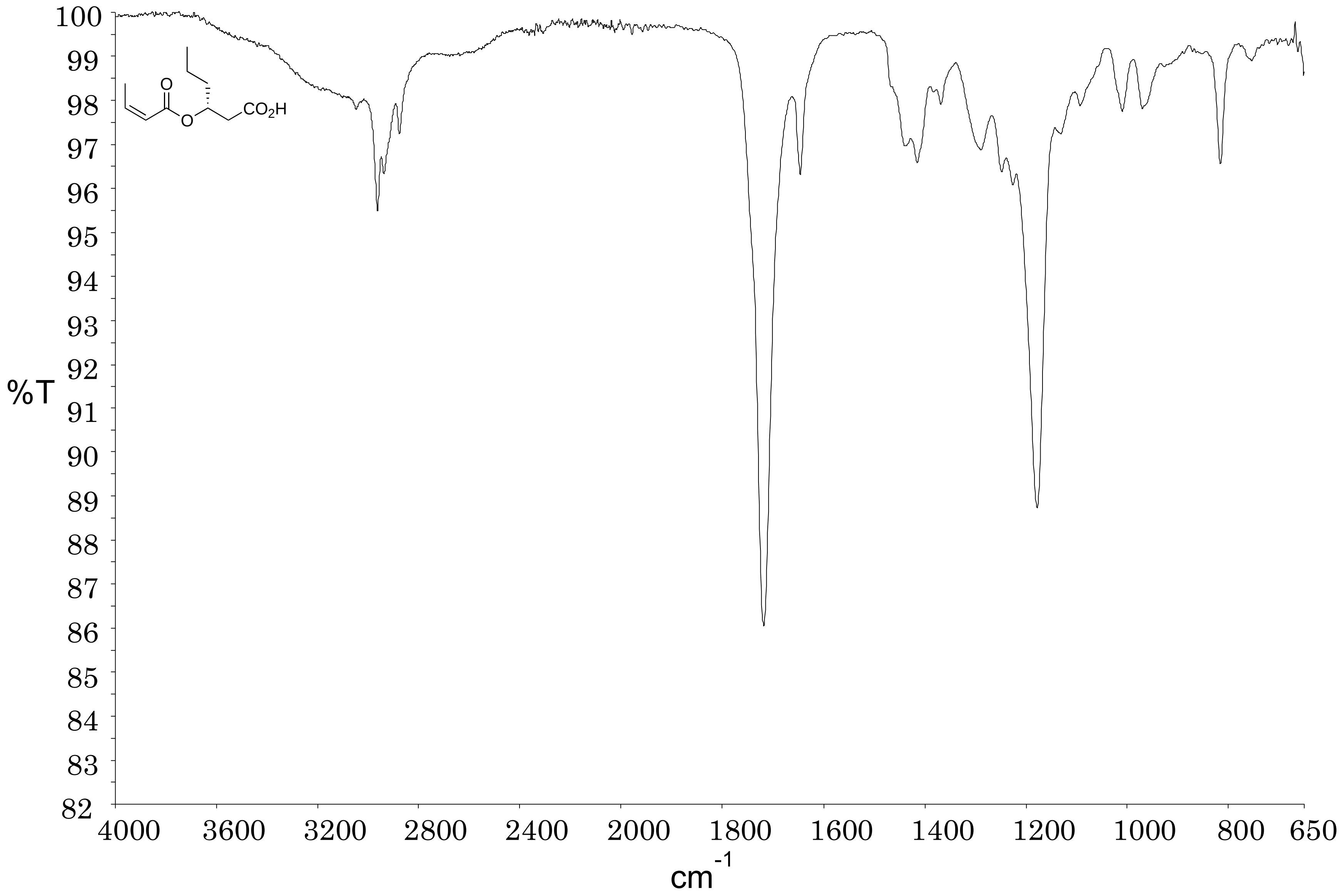

IR spectrum of **2**

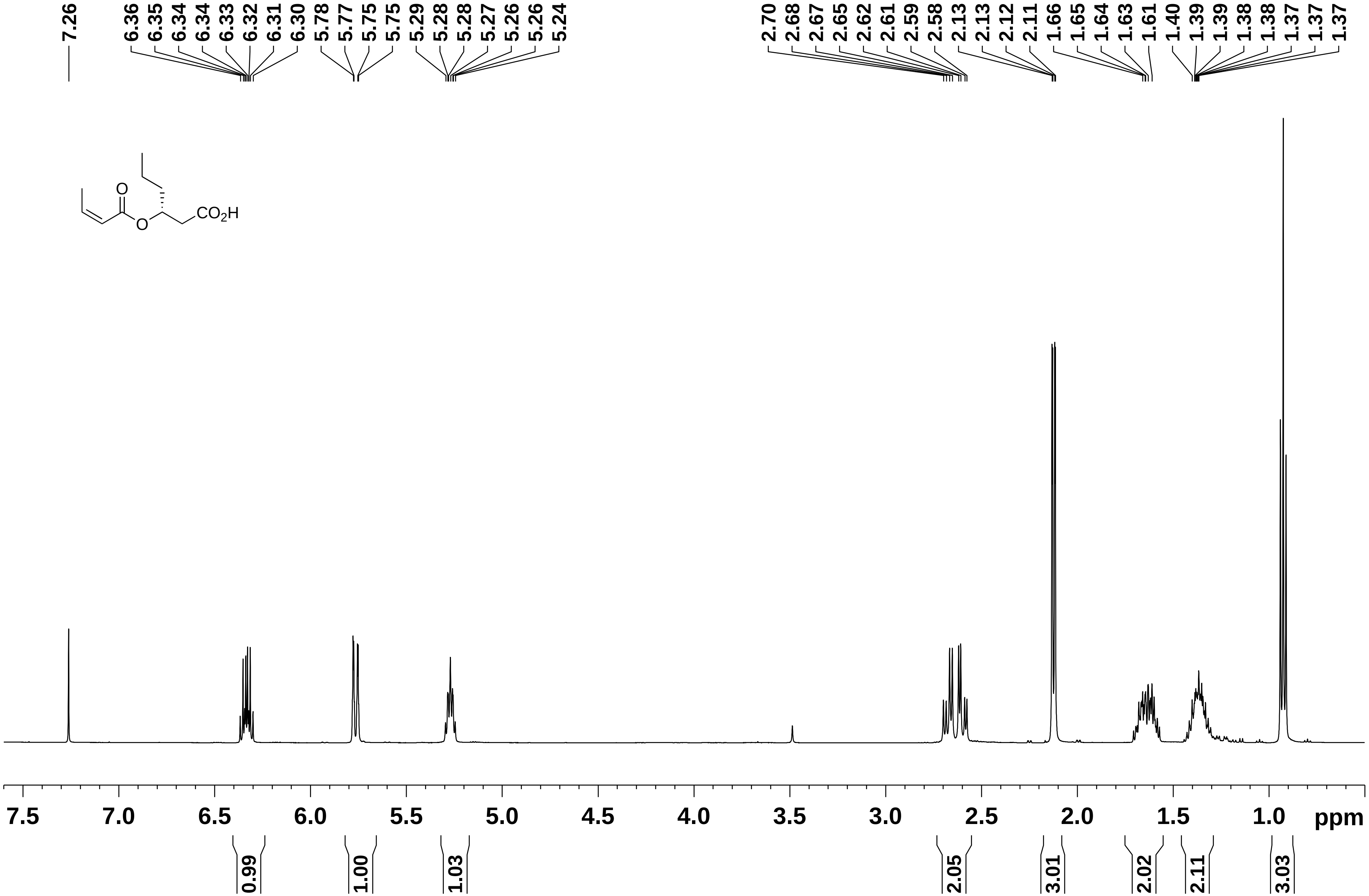

^{1}H NMR spectrum of **2** ($CDCl_3$, 500 MHz)

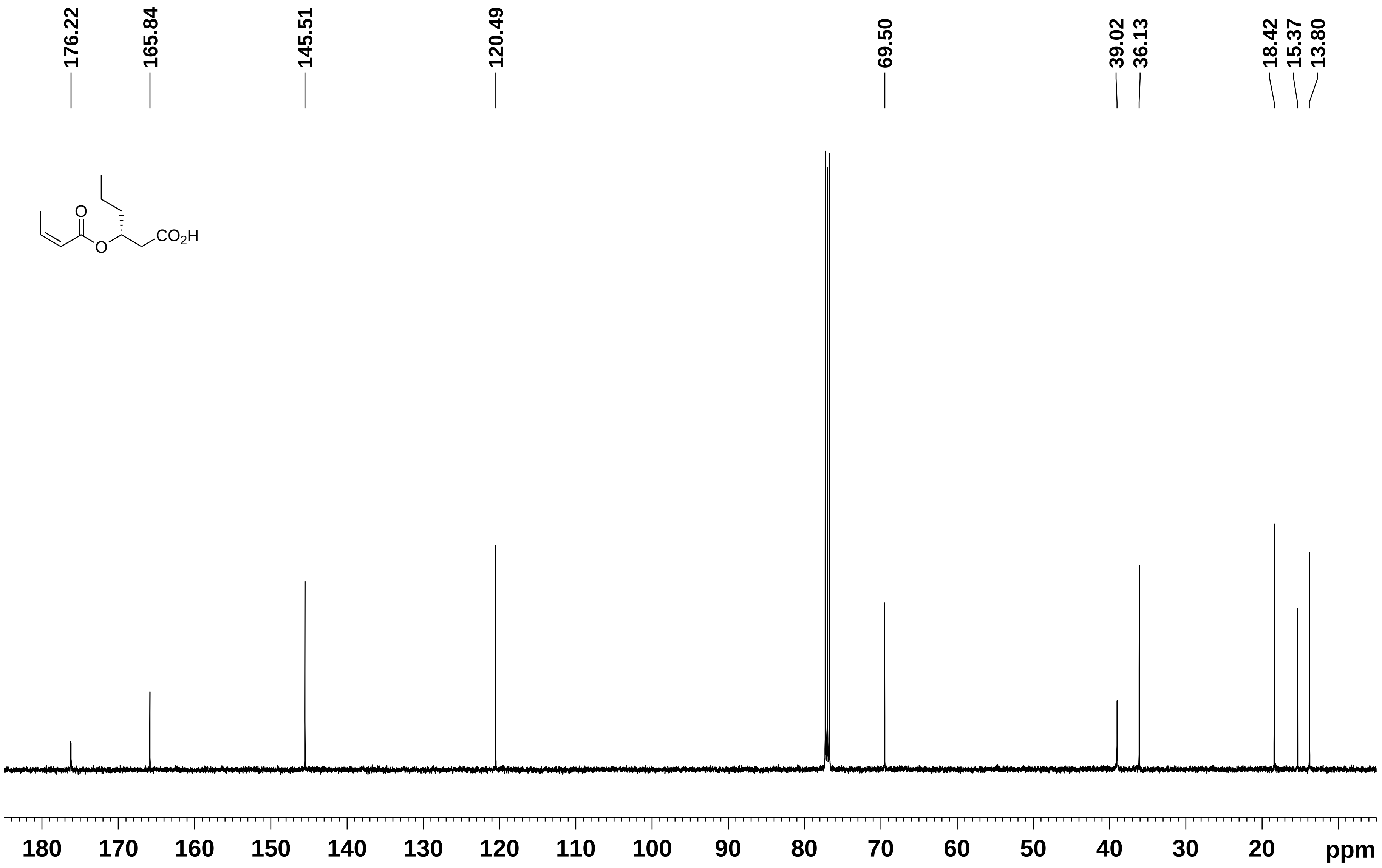

^{13}C NMR spectrum of **2** ($CDCl_3$, 500 MHz)

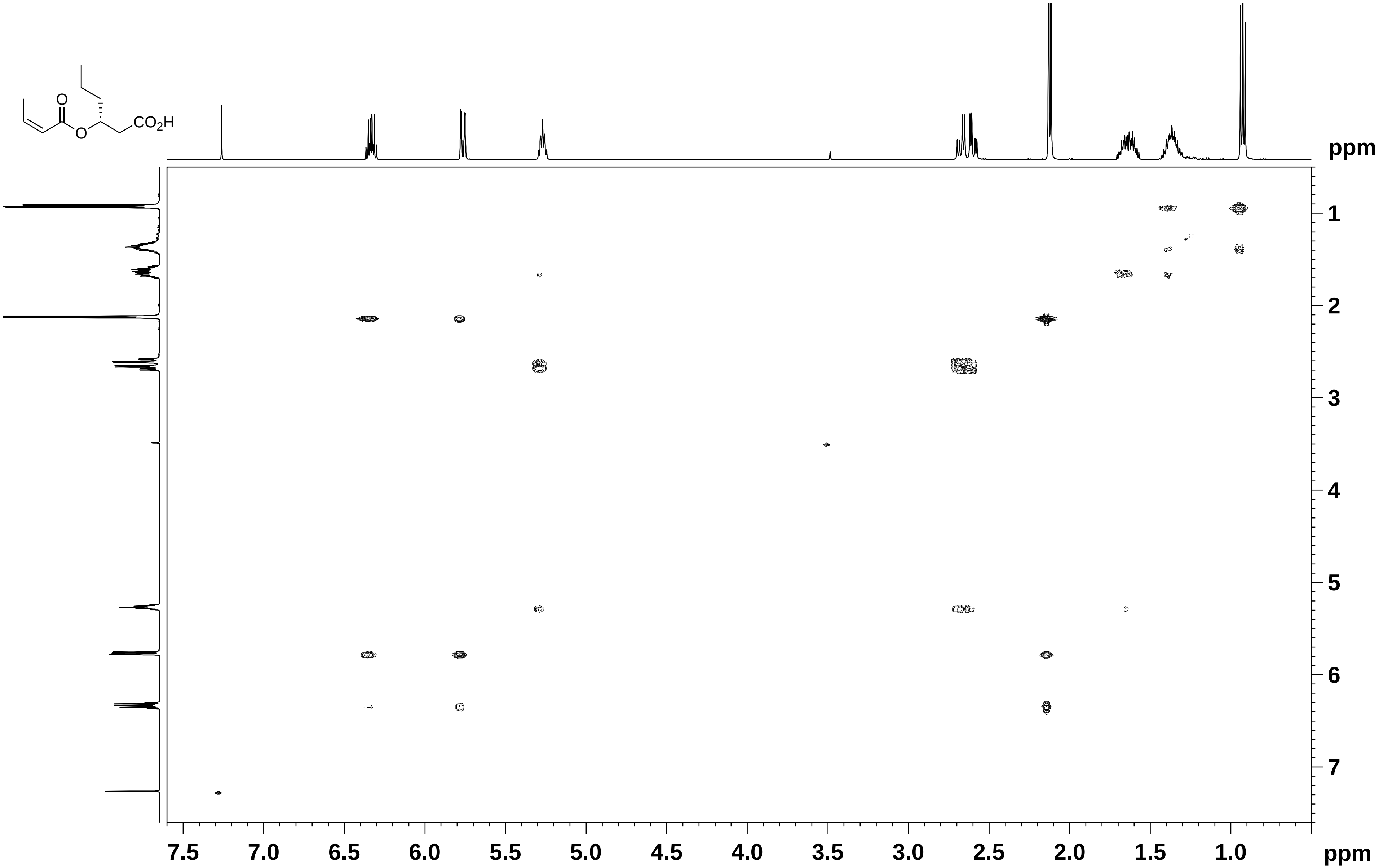

COSY spectrum of **2** ($CDCl_3$, 500 MHz).

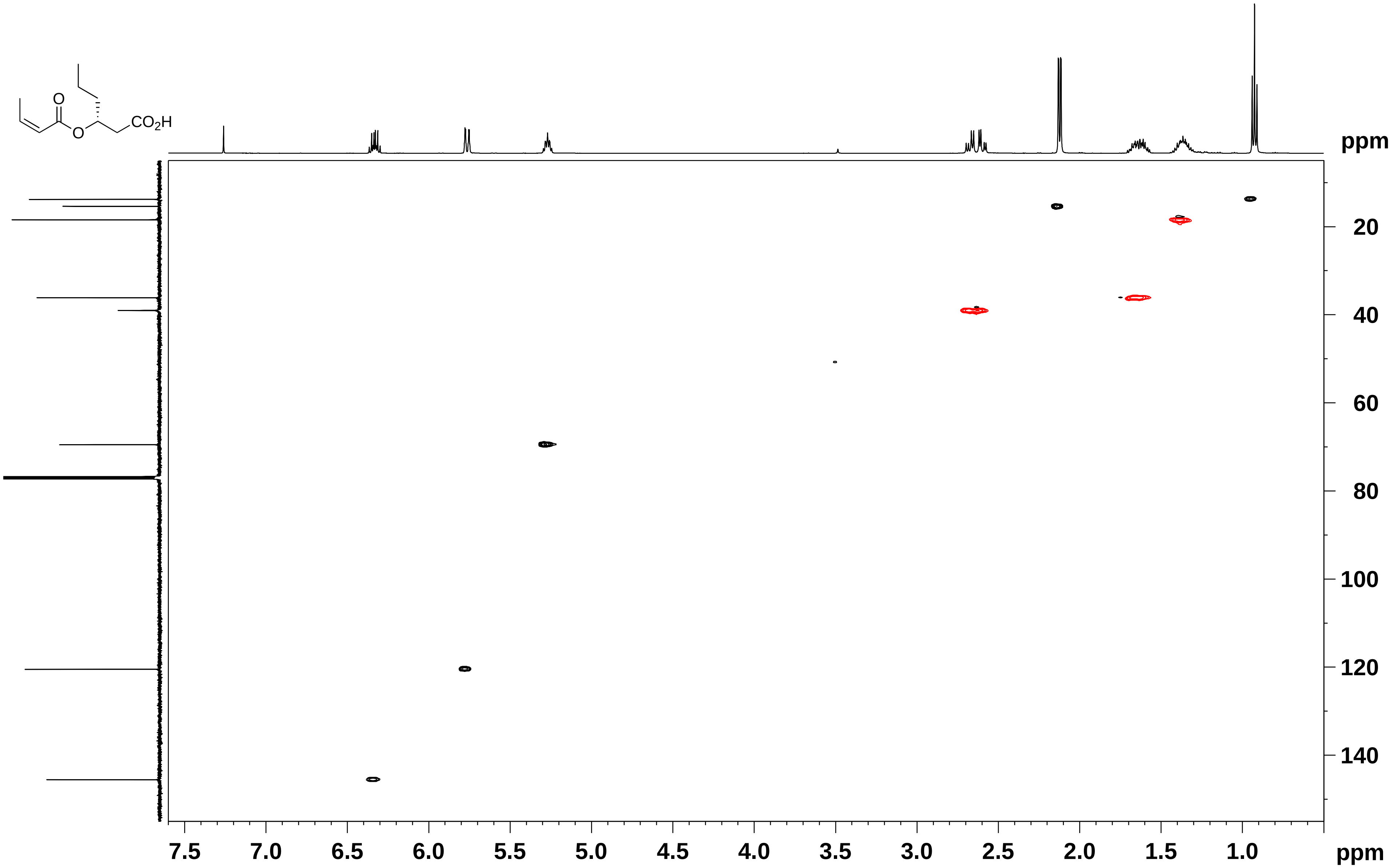

HSQC spectrum of **2** ($CDCl_3$, 500 MHz)

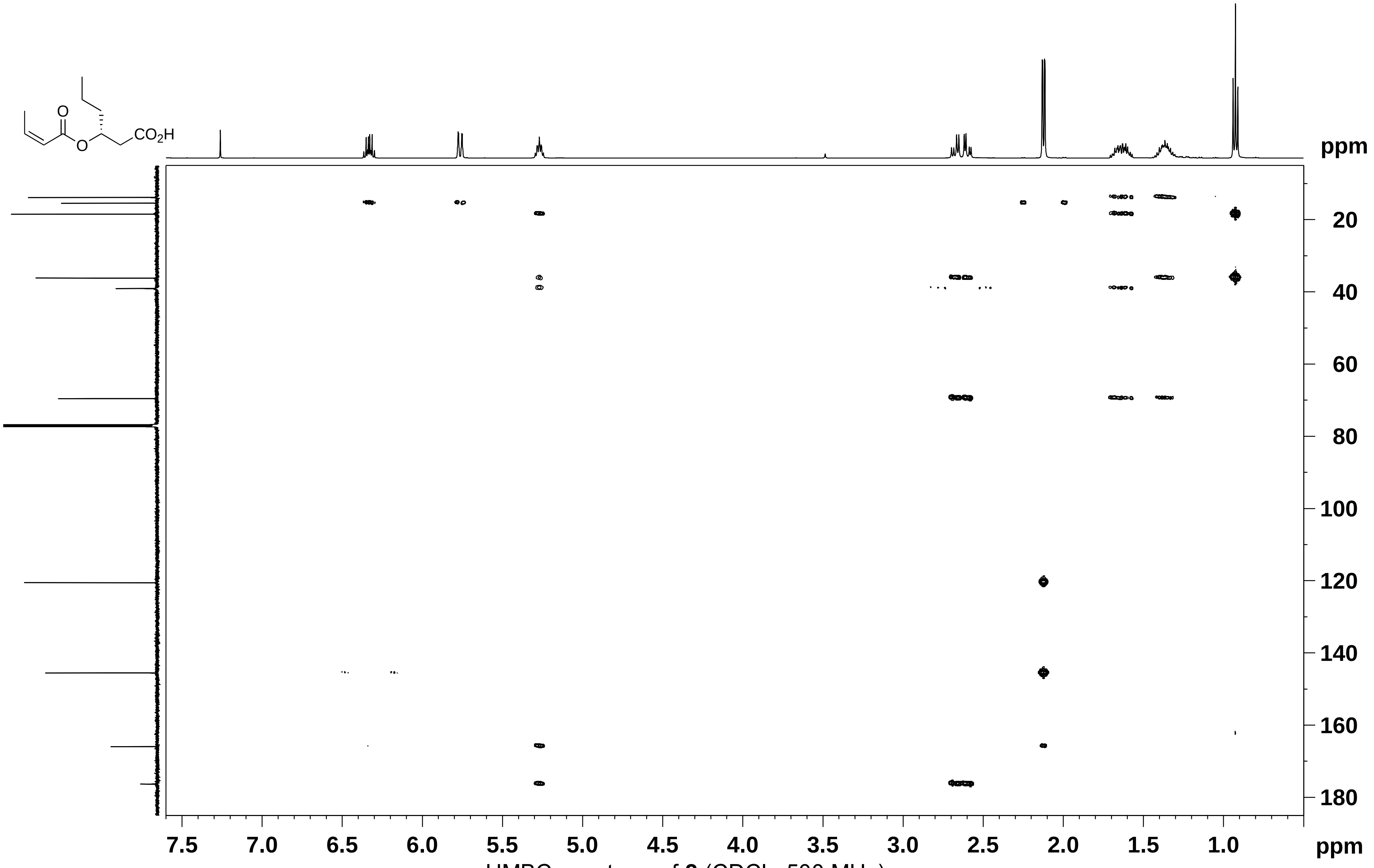

HMBC spectrum of **2** ($CDCl_3$, 500 MHz)

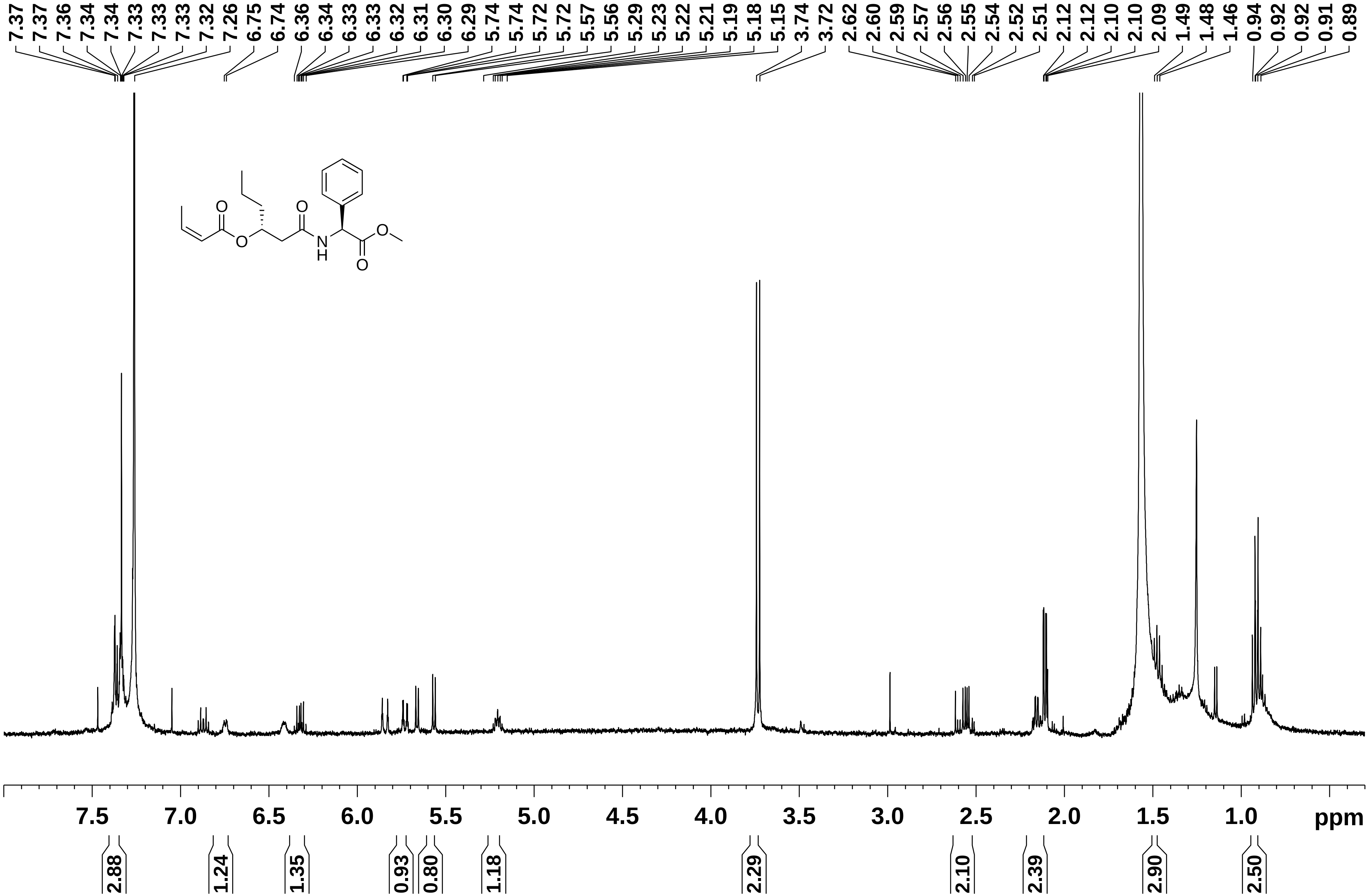

^{1}H NMR spectrum of (*S*)-PGME amide (**2a**) ($CDCl_3$, 500 MHz)

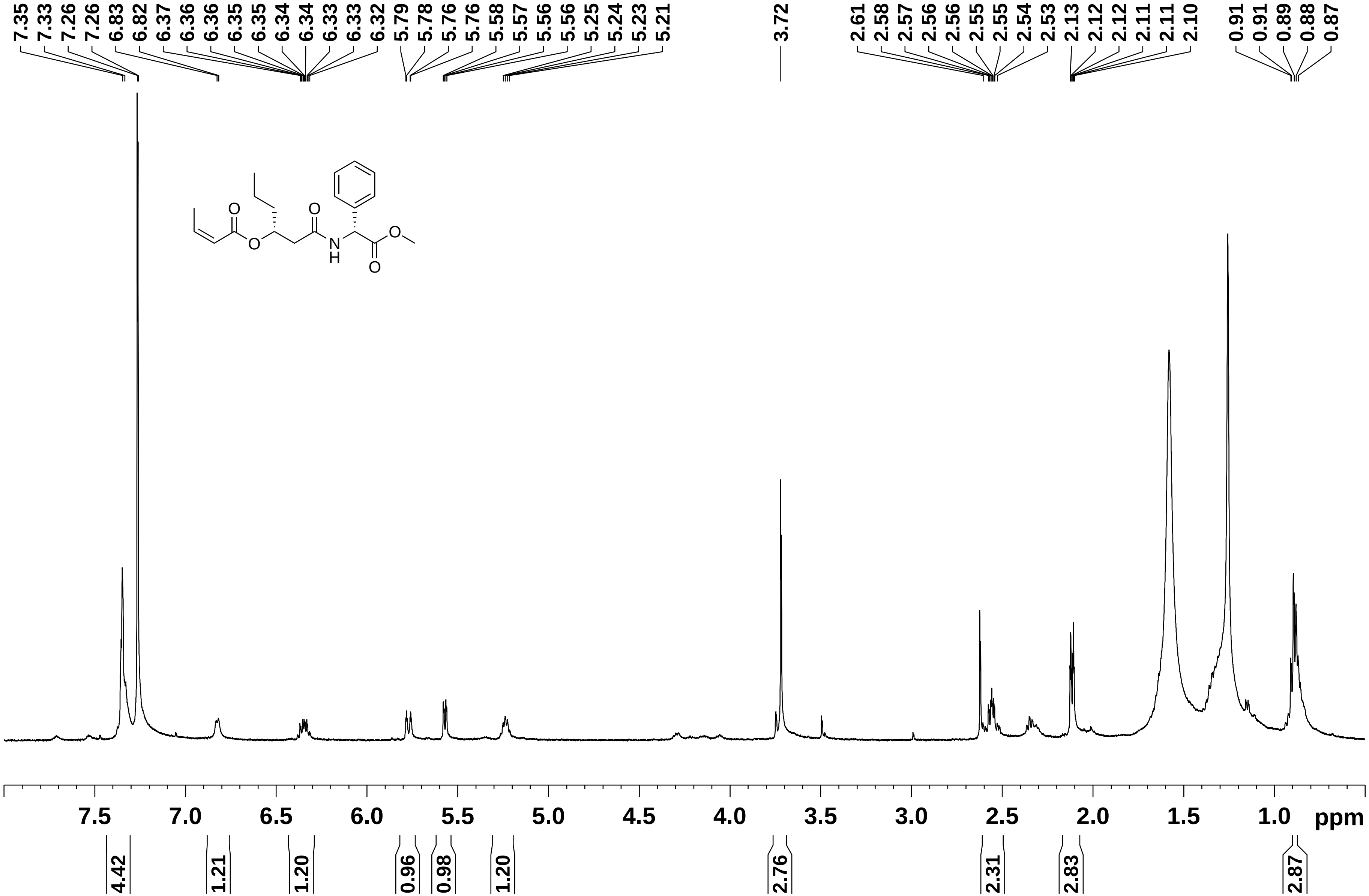

^{1}H NMR spectrum of (*R*)-PGME amide (**2b**) ($CDCl_3$, 500 MHz)

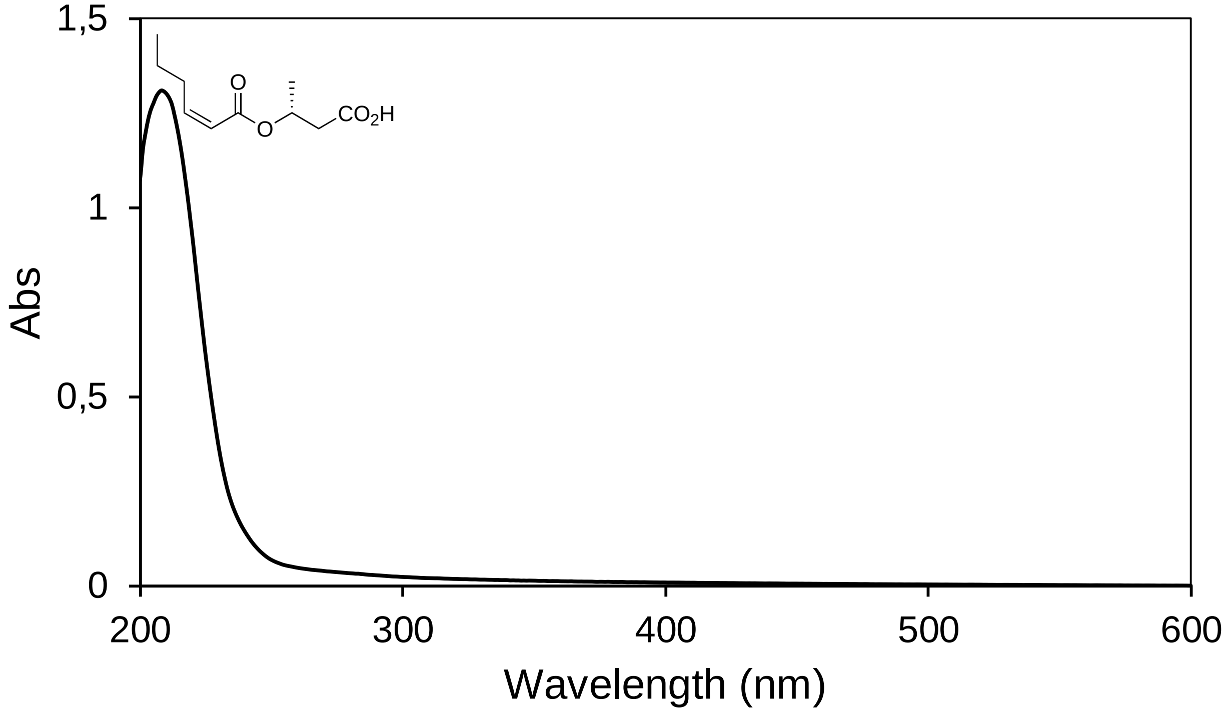

UV spectrum of **3**

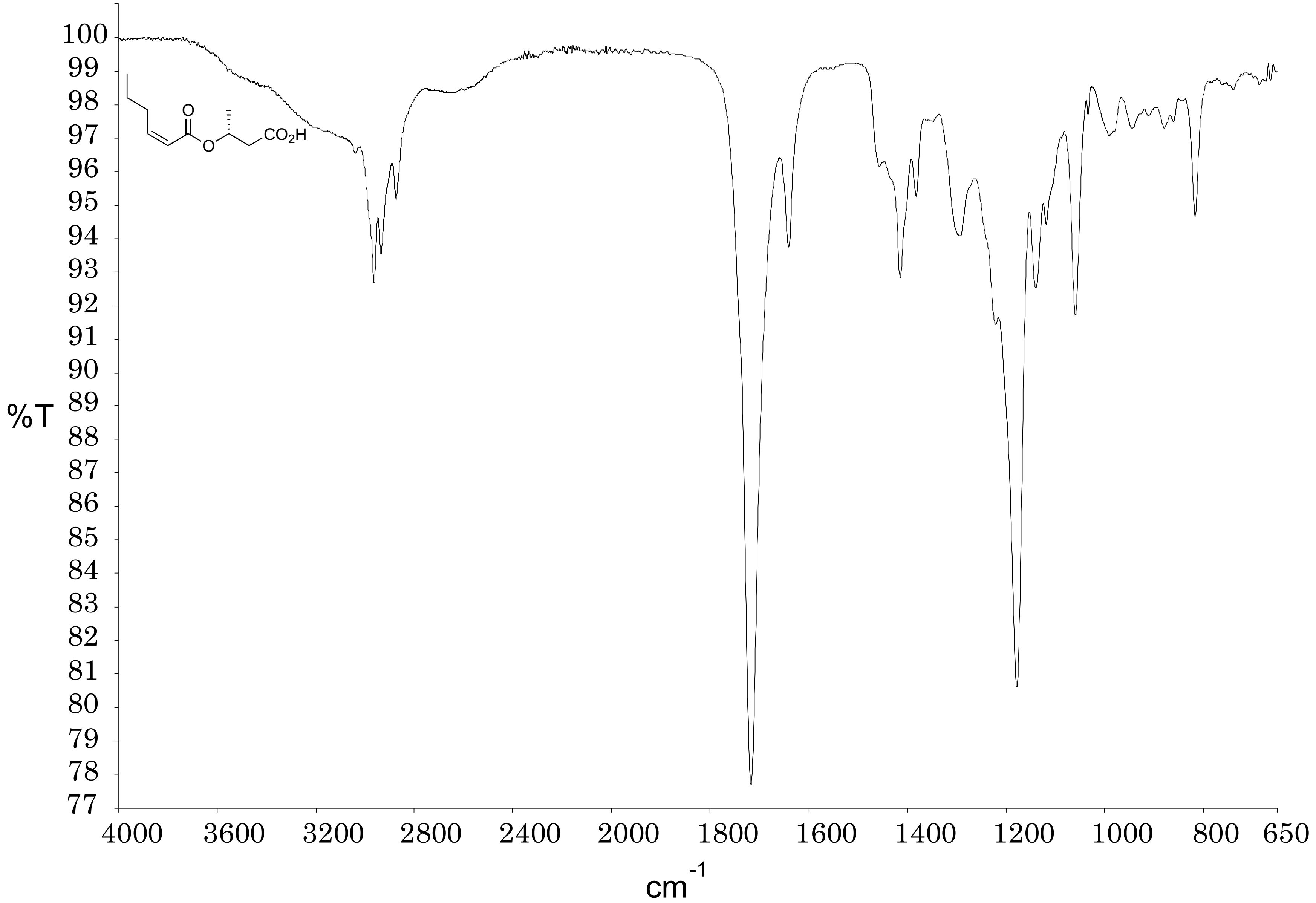

IR spectrum of **3**

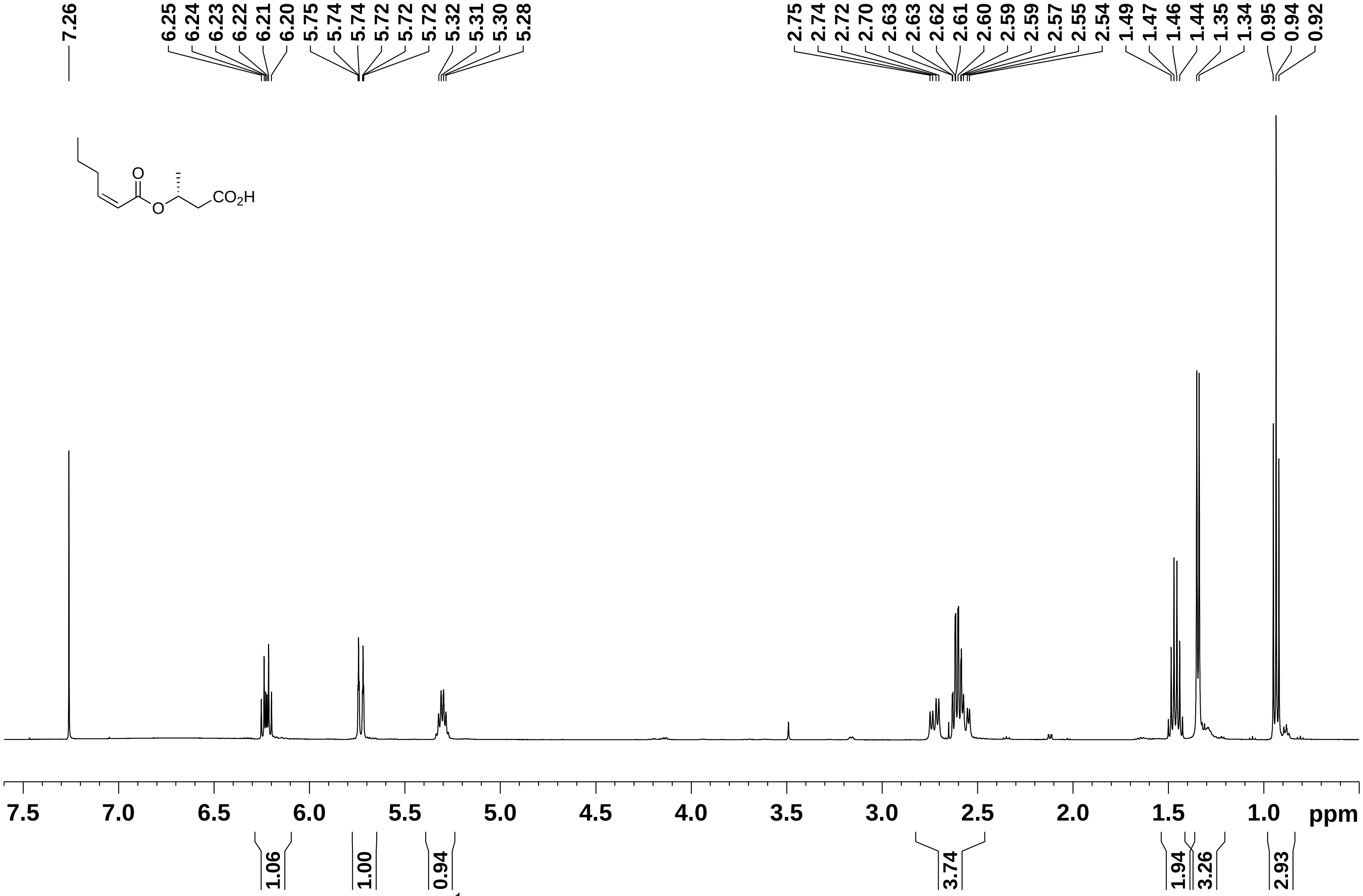

^{1}H NMR spectrum of **3** ($CDCl_3$, 500 MHz)

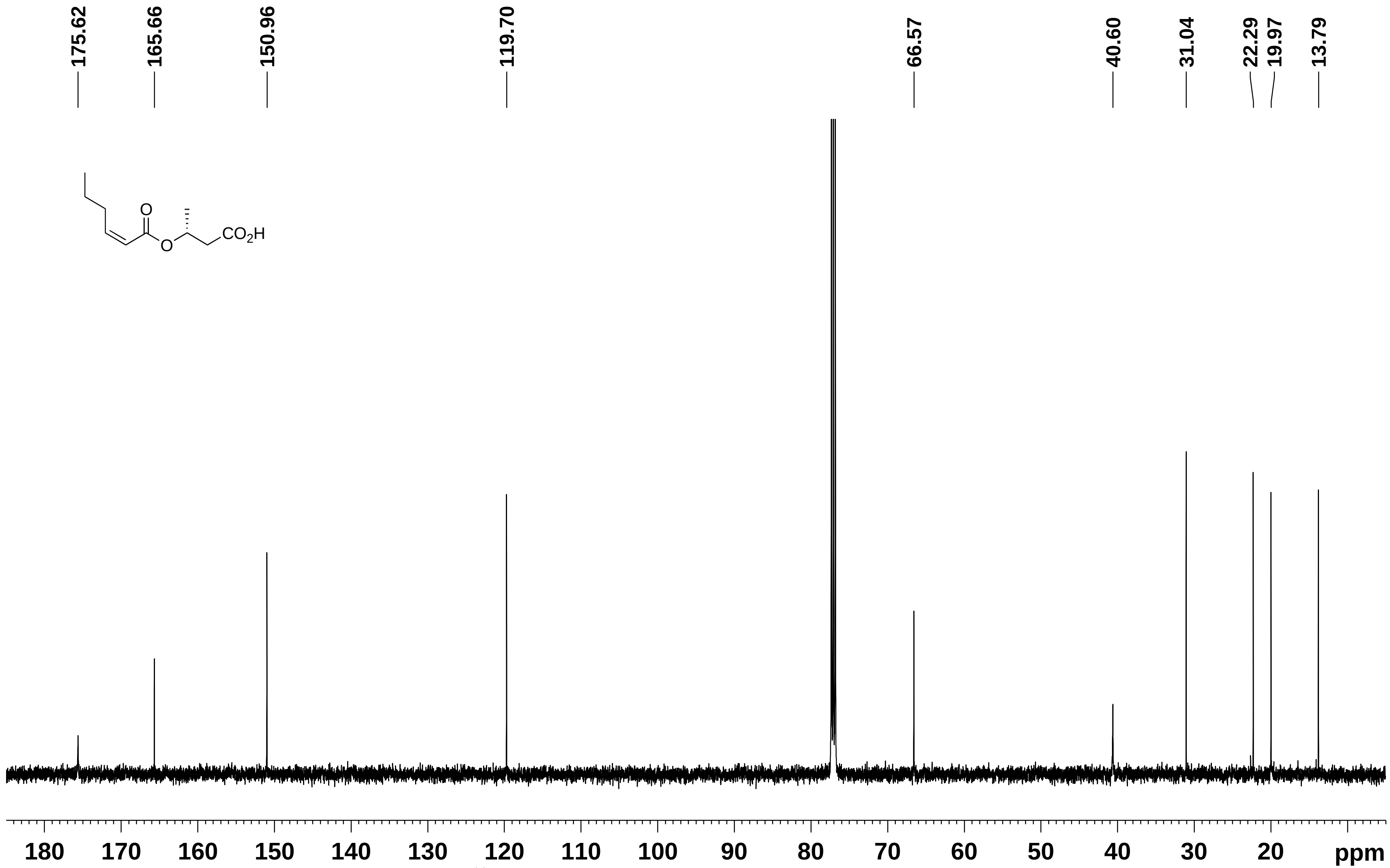

^{13}C NMR spectrum of **3** ($CDCl_3$, 125 MHz)

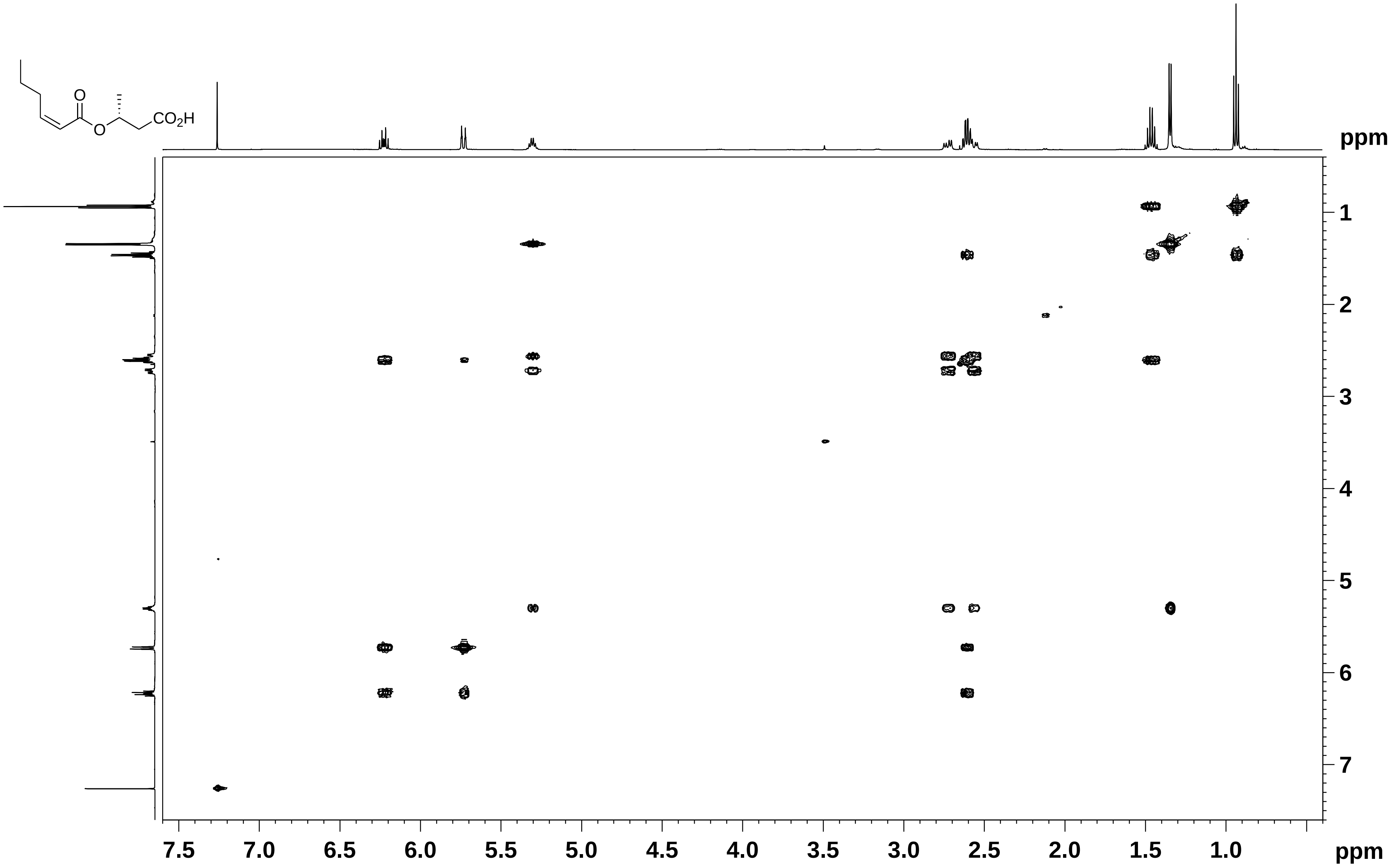

COSY spectrum of **3** ($CDCl_3$, 500 MHz)

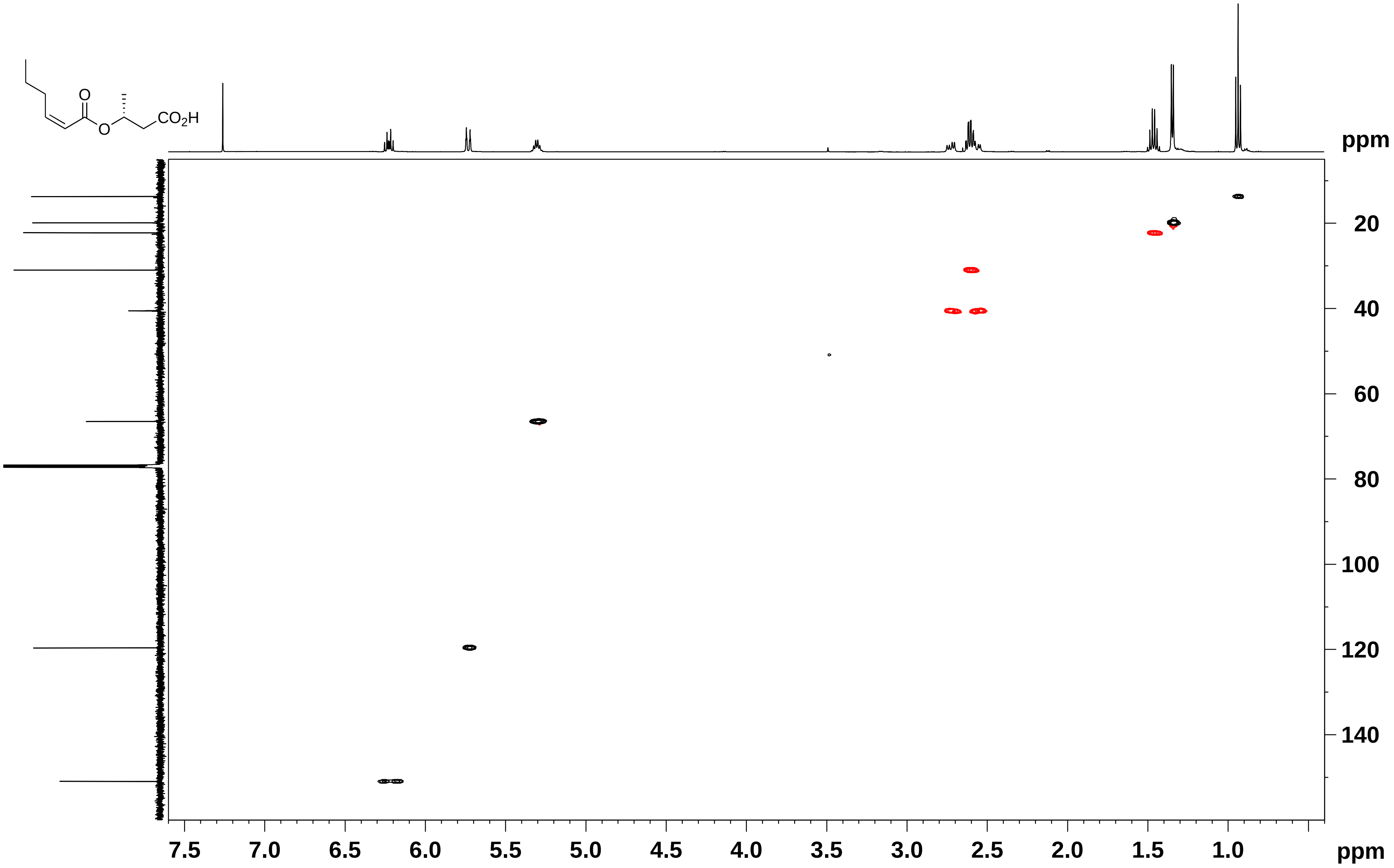

HSQC spectrum of **3** ($CDCl_3$, 500 MHz)

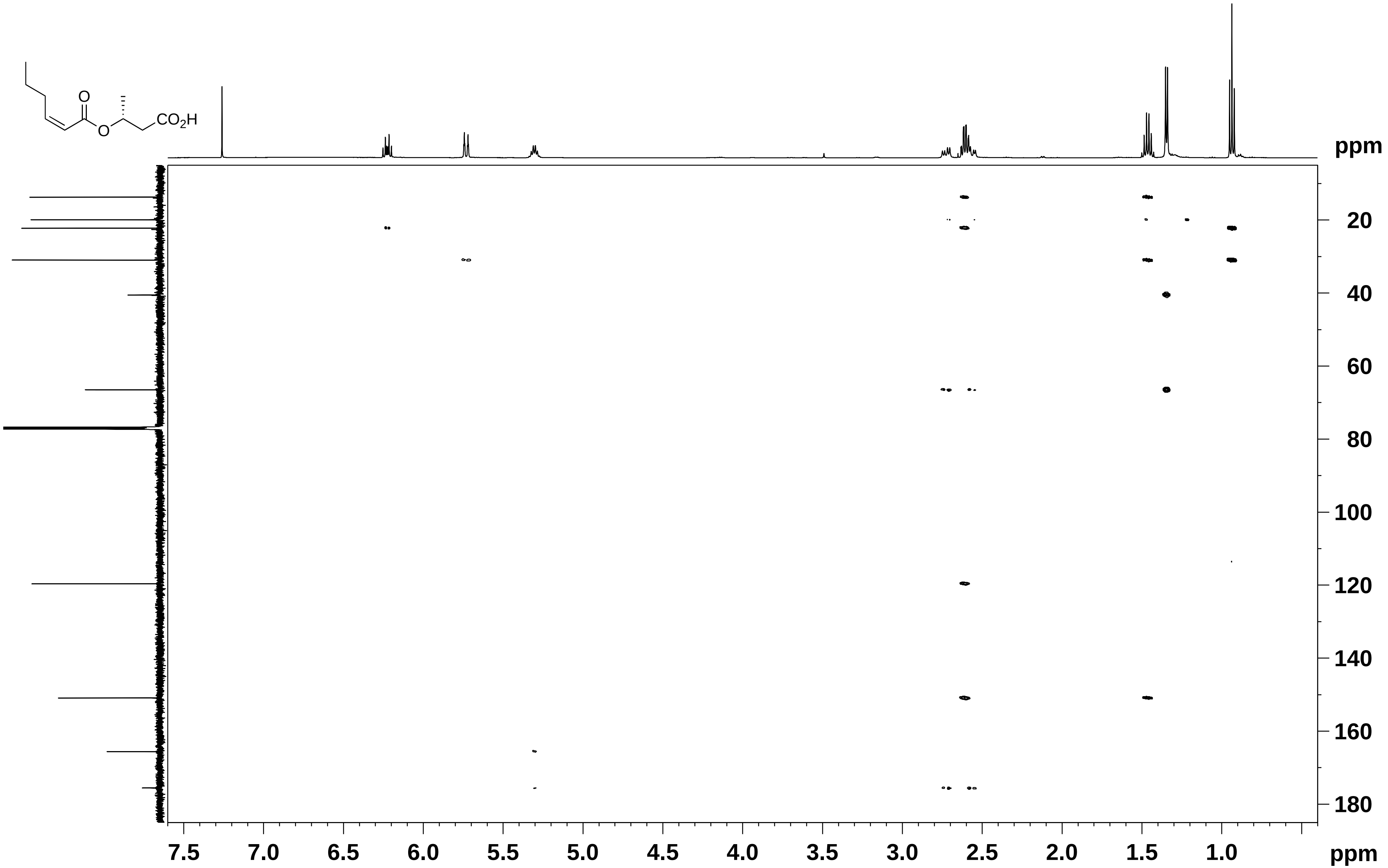

HMBC spectrum of **3** ($CDCl_3$, 500 MHz)

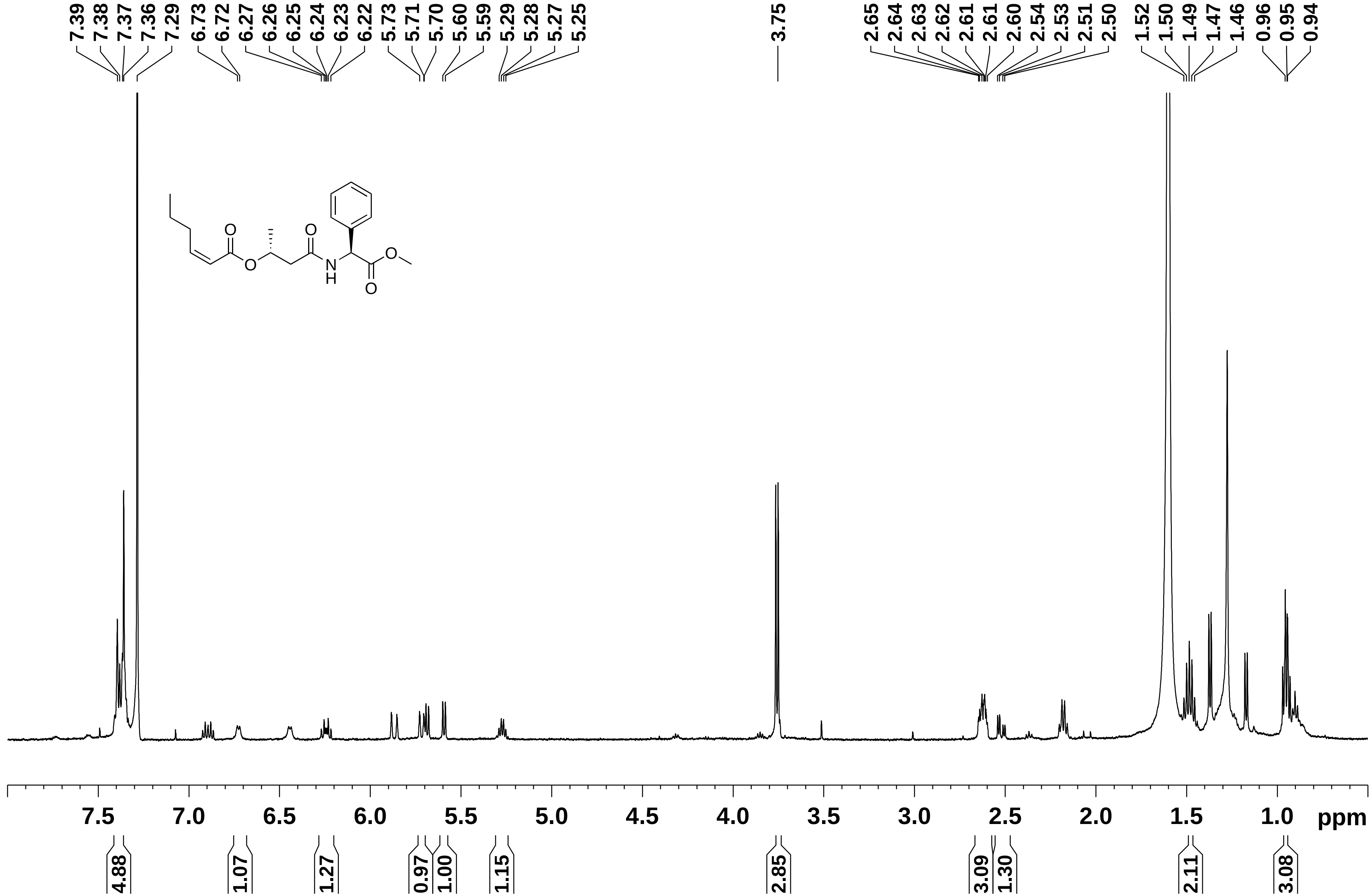

^{1}H NMR spectrum of (*S*)-PGME amide (**3a**) ($CDCl_3$, 500 MHz)

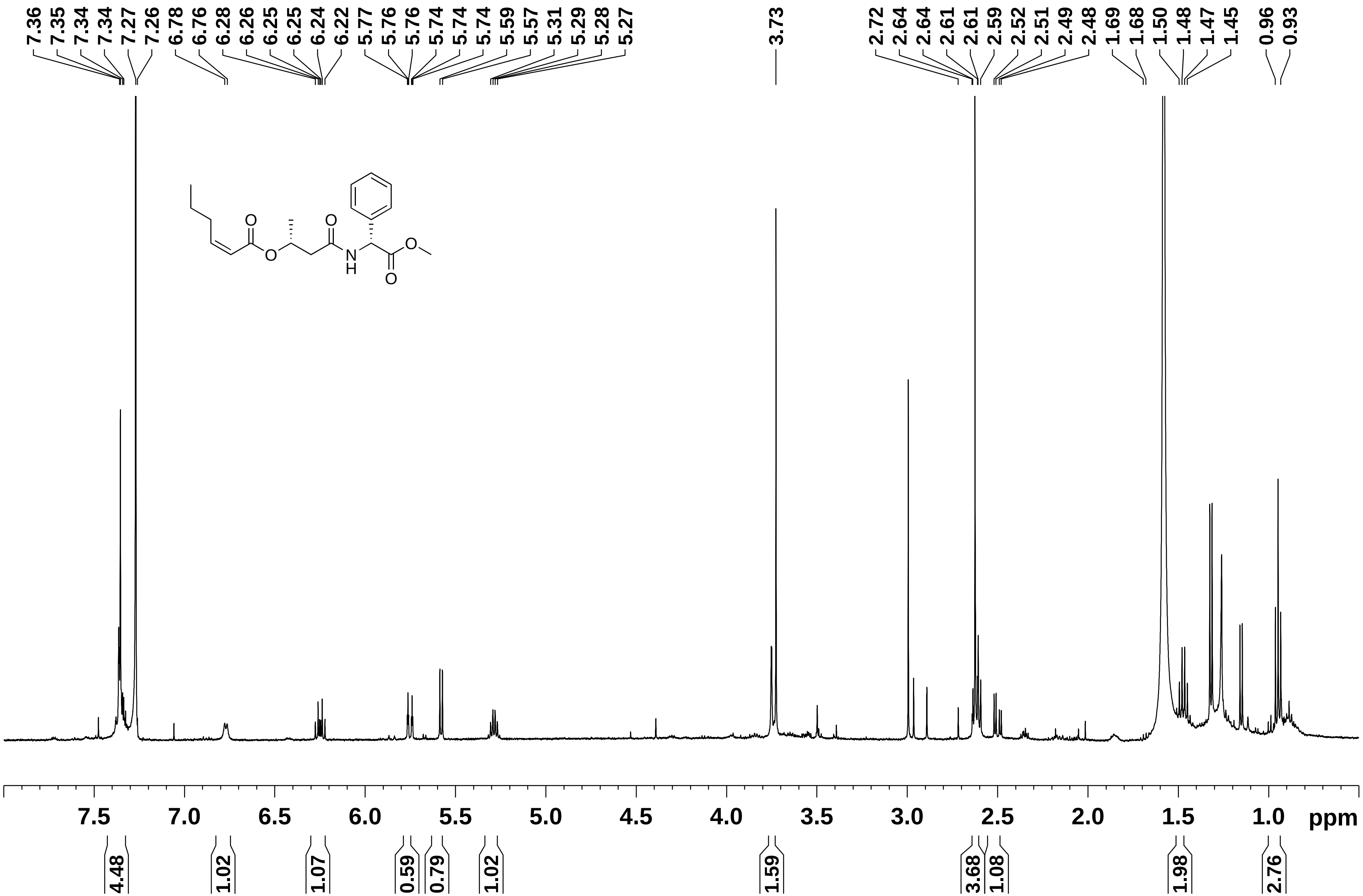

^{1}H NMR spectrum of (*R*)-PGME amide (**3b**) ($CDCl_3$, 500 MHz)

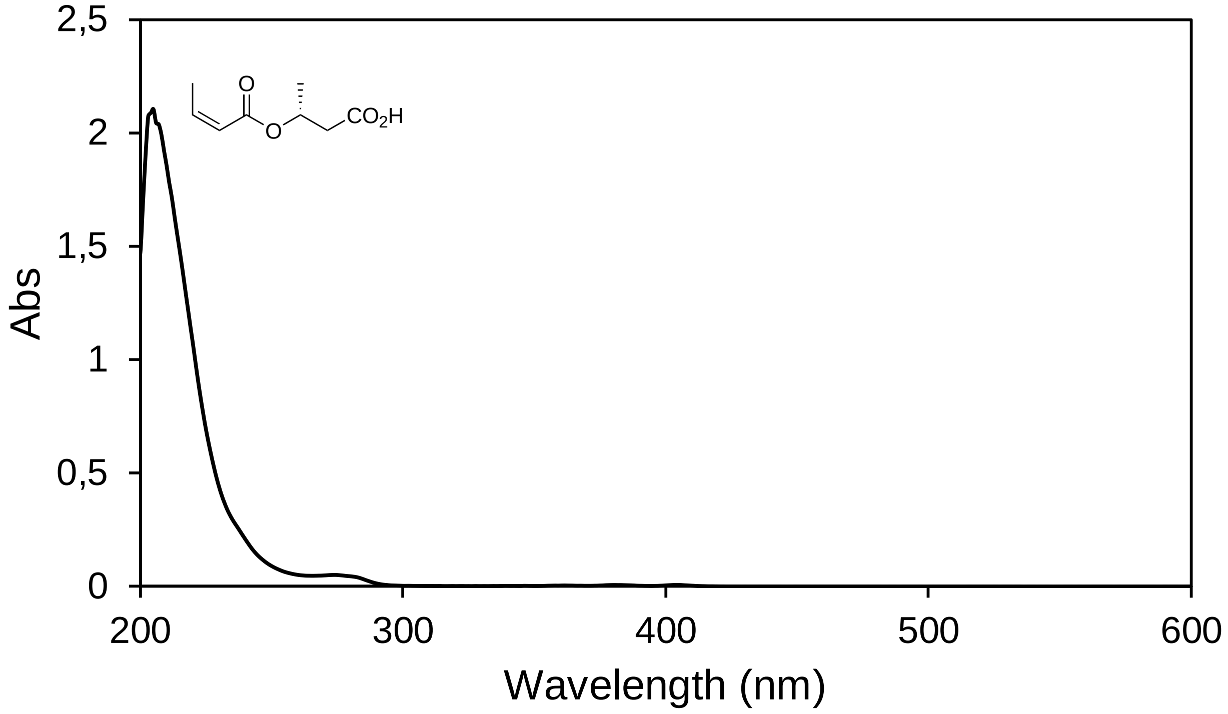

UV spectrum of **4**

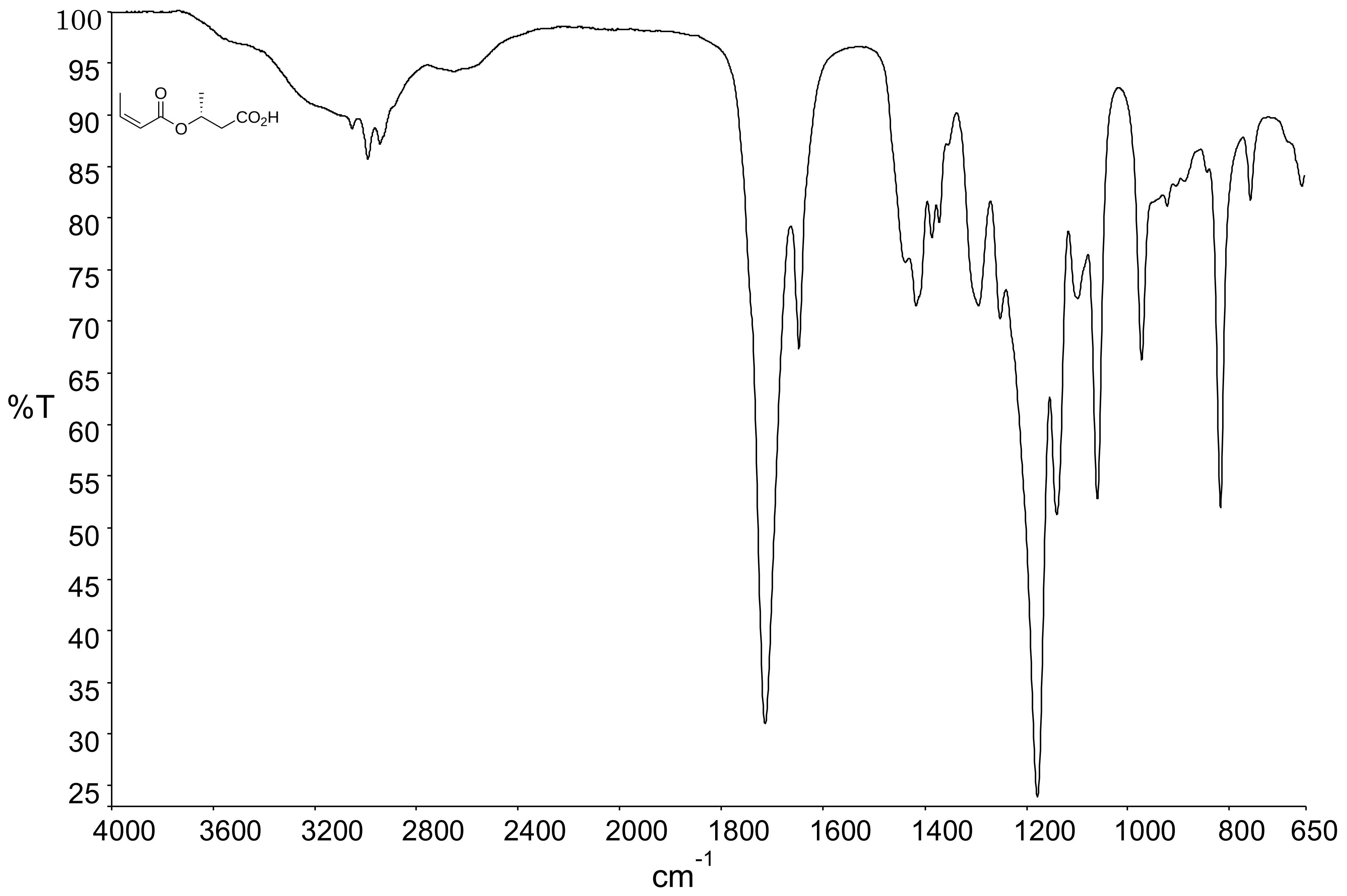

UV spectrum of **4**

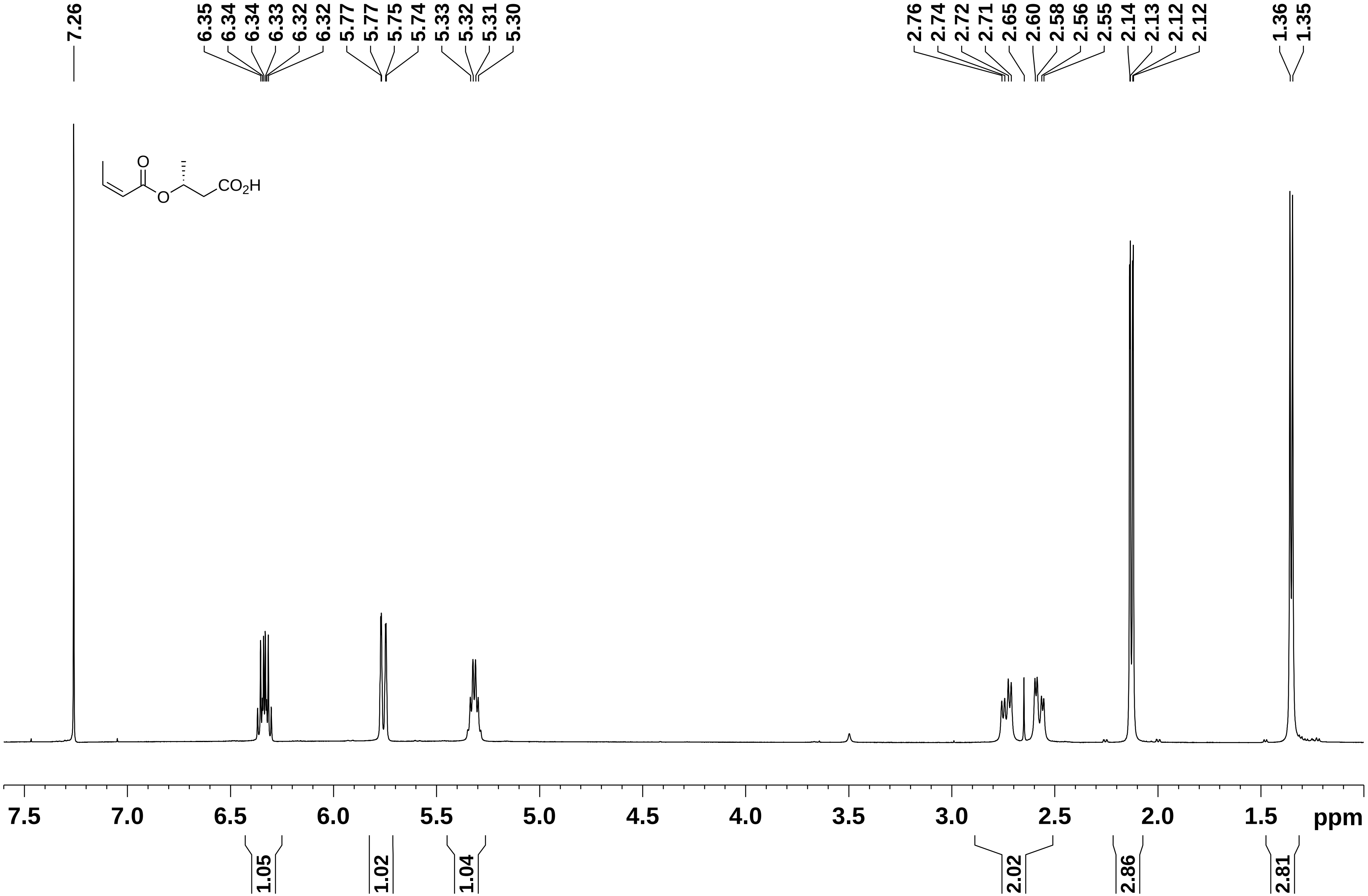

^1H NMR spectrum of **4** ($CDCl_3$, 500 MHz)

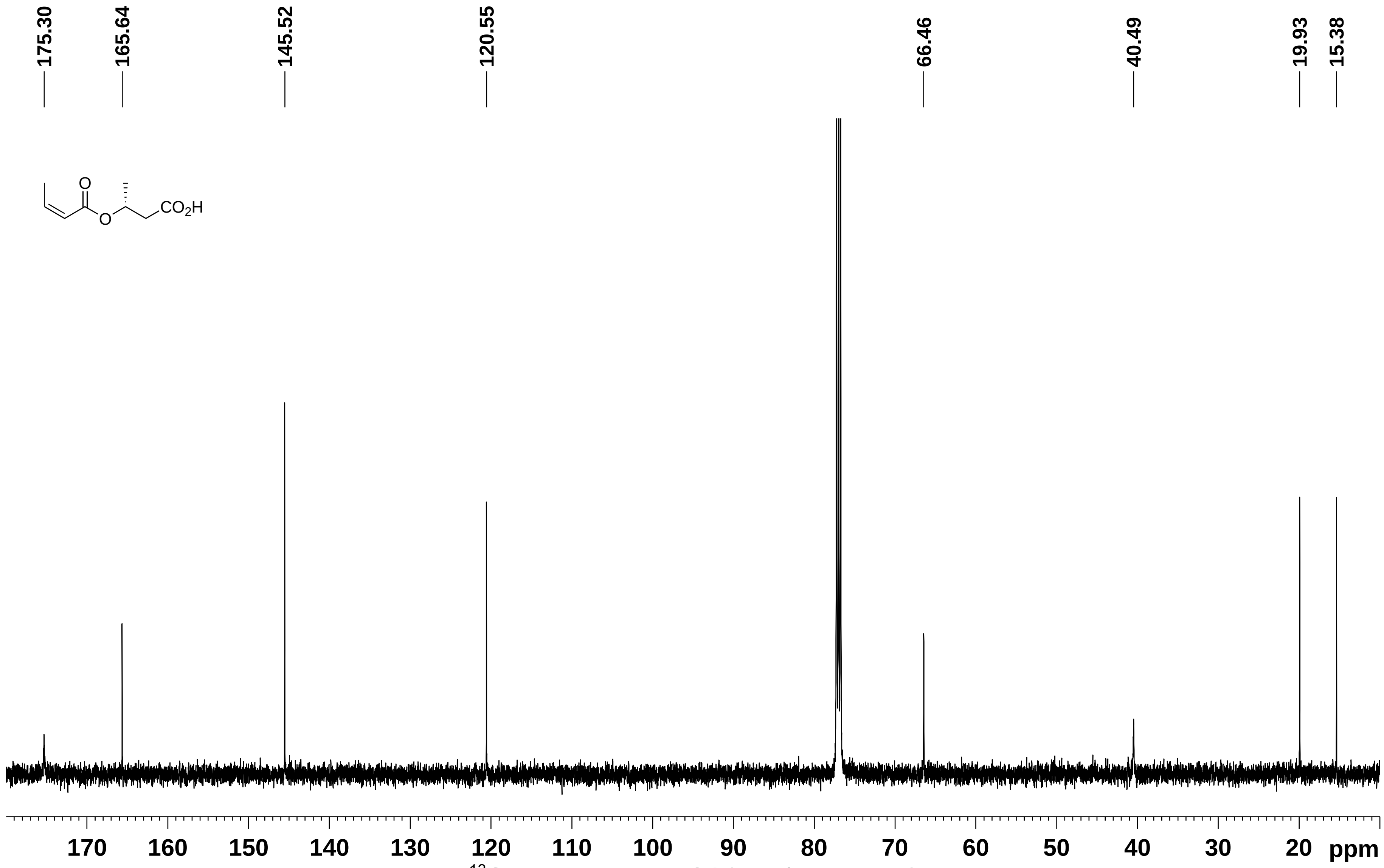

^{13}C NMR spectrum of **4** ($CDCl_3$, 500 MHz)

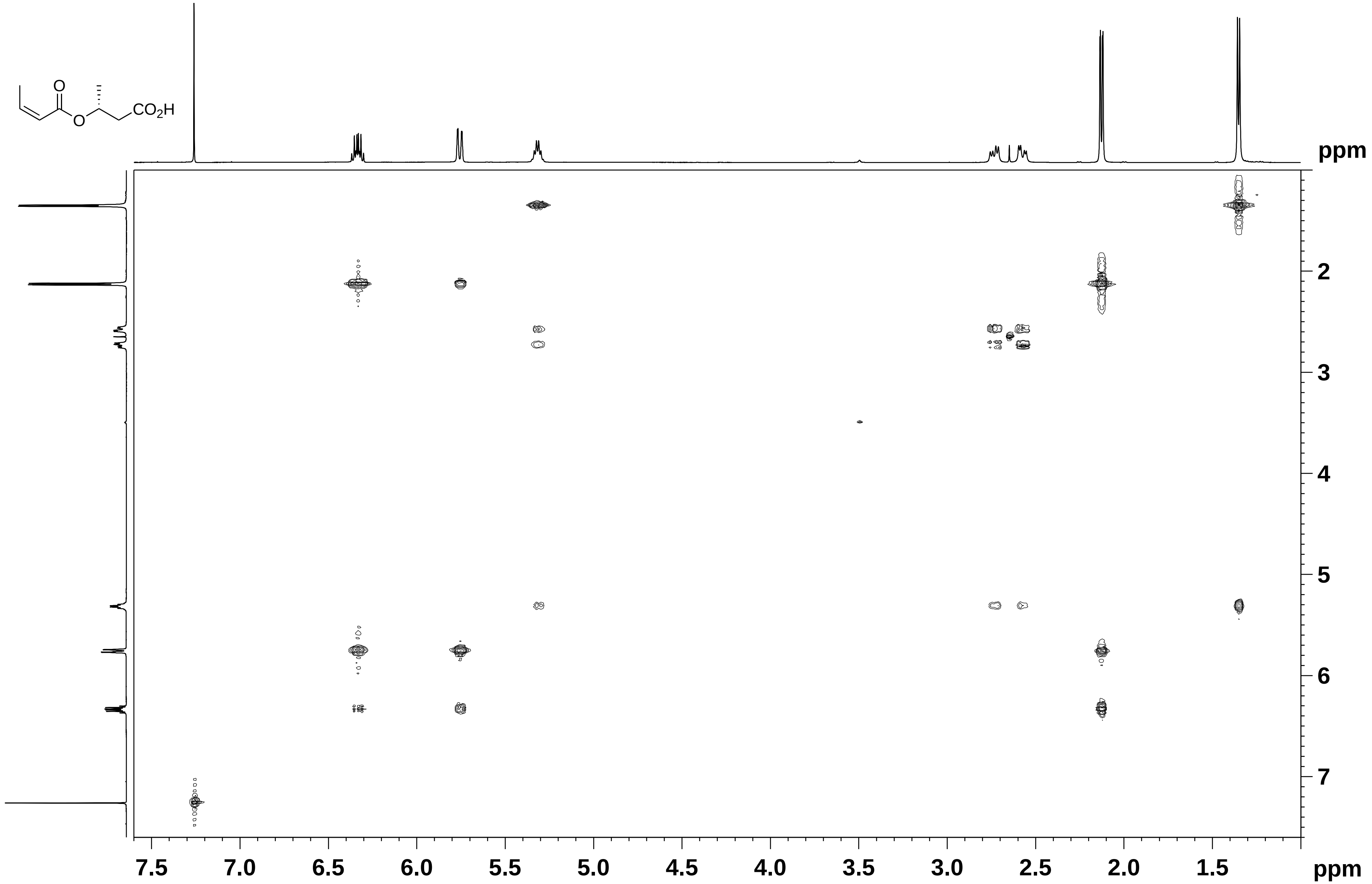

COSY spectrum of **4** ($CDCl_3$, 500 MHz)

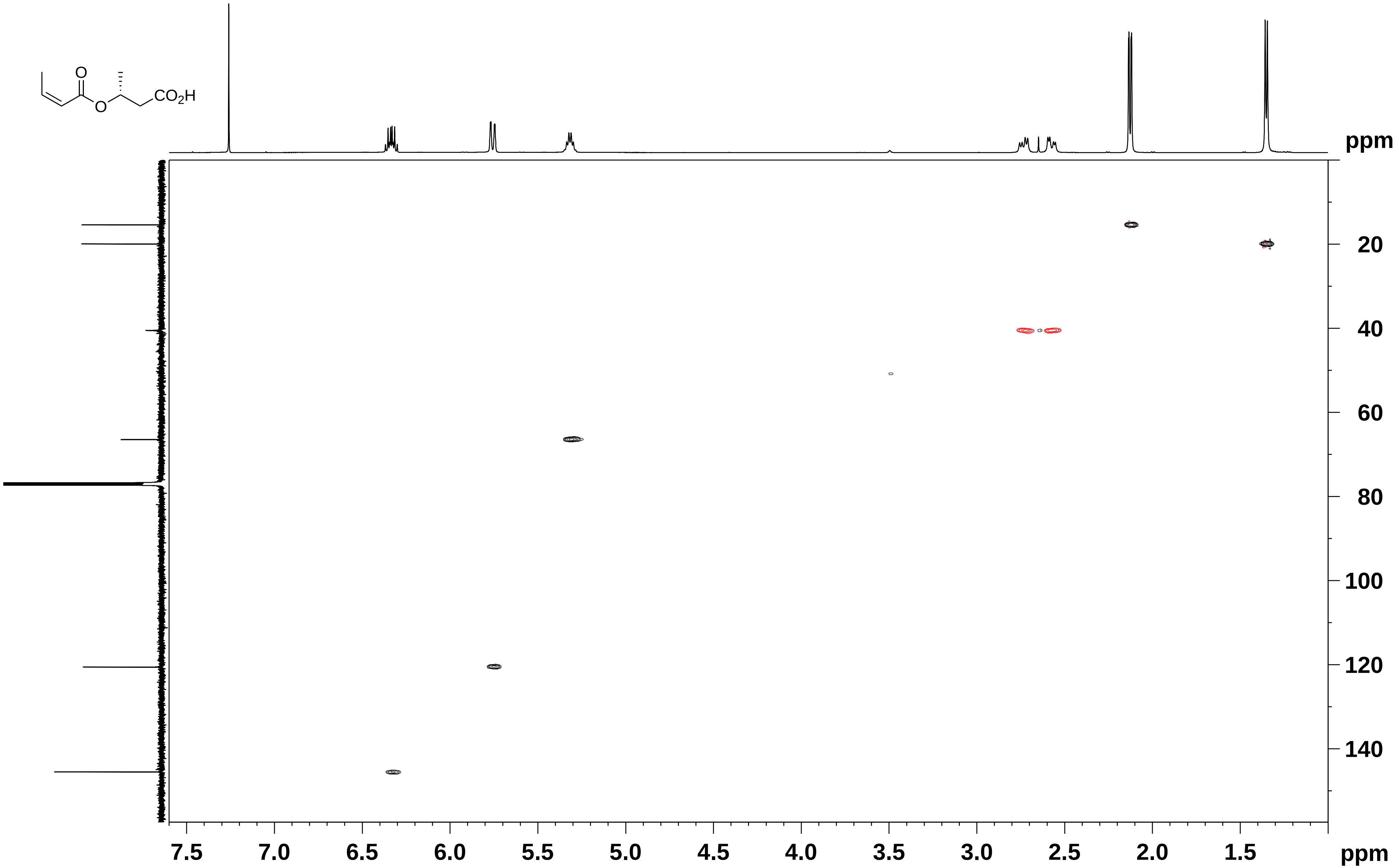

HSQC spectrum of **4** ($CDCl_3$, 500 MHz)

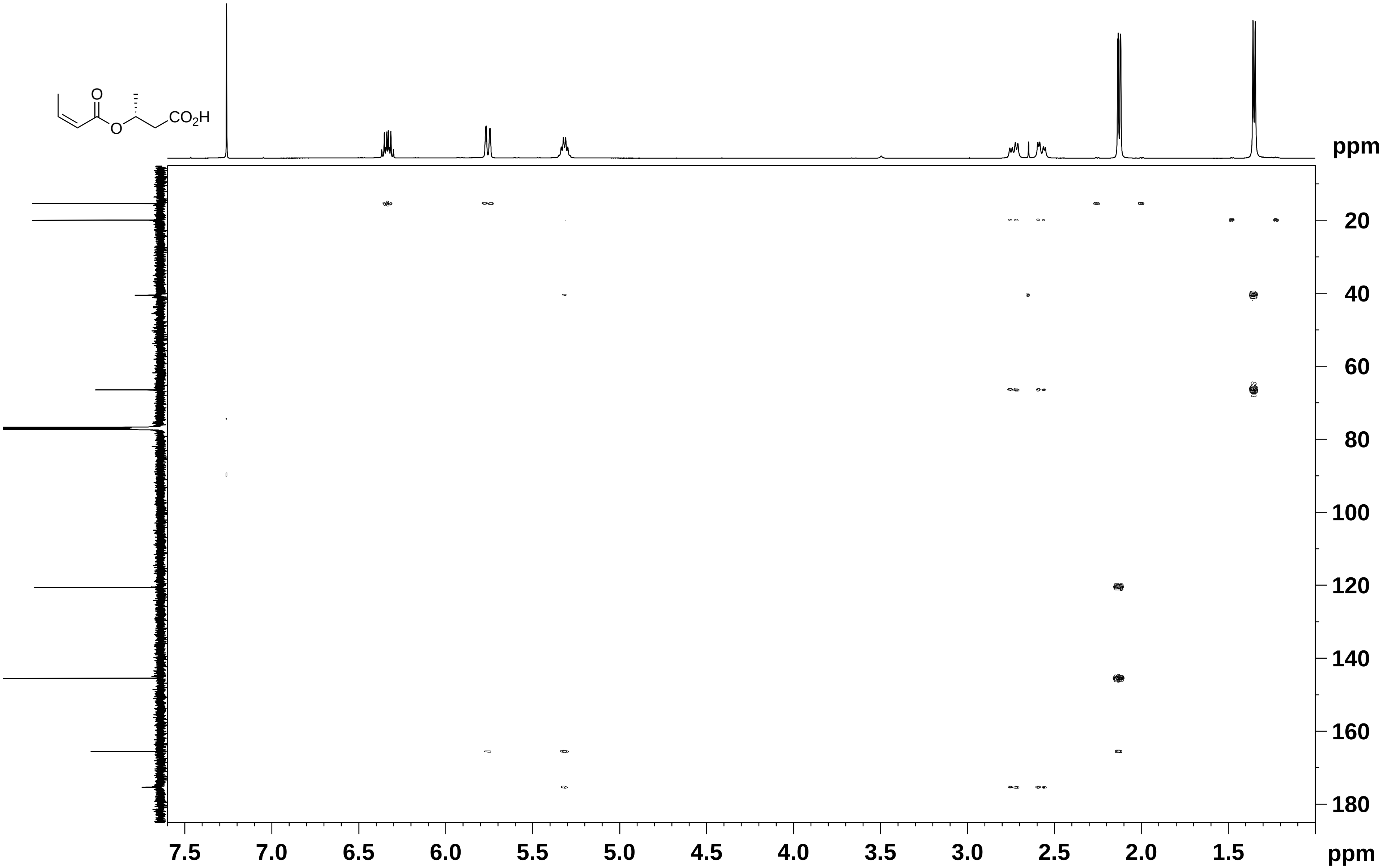

HMBC spectrum of **4** ($CDCl_3$, 500 MHz)

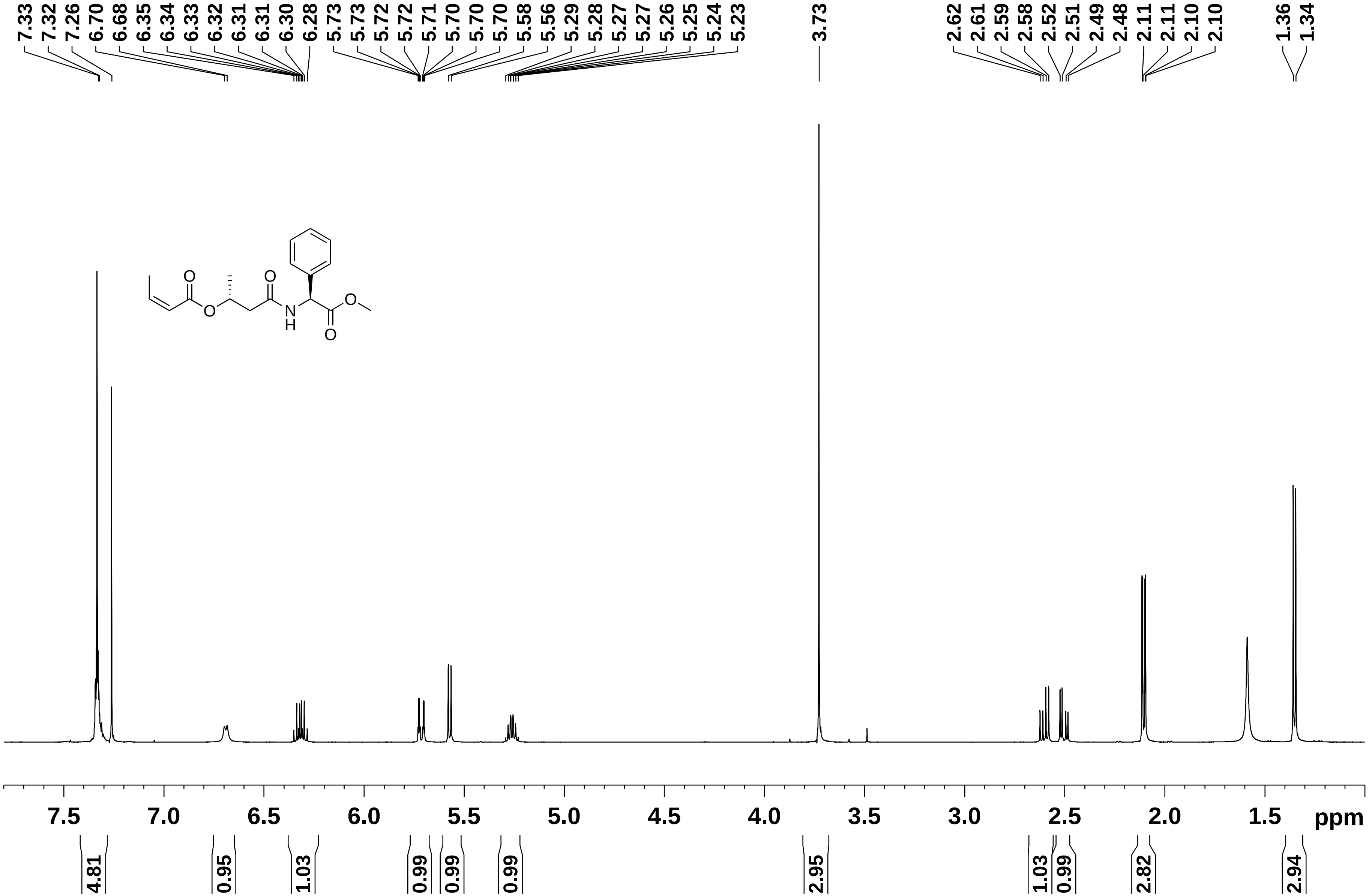

^{1}H NMR spectrum of (*S*)-PGME amide (**4a**) ($CDCl_3$, 500 MHz)

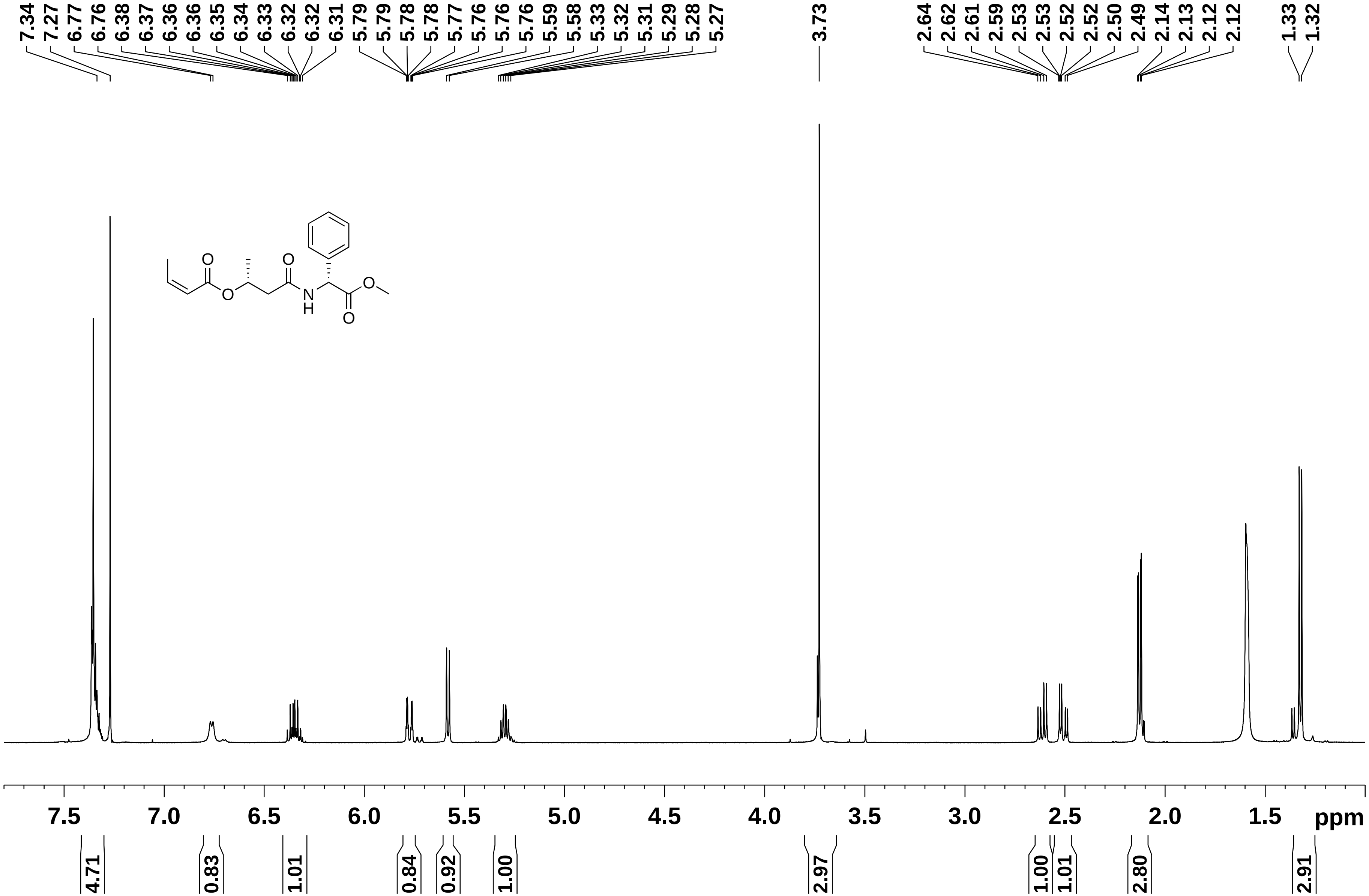

^{1}H NMR spectrum of (*R*)-PGME amide (**4b**) ($CDCl_3$, 500 MHz)

Compound characterization data

(*R*)-*O*-Isocrotonyl-3-hydroxypentanoic acid (**1**): colorless amorphous solid; $[\alpha]^{24}_{D}$ -53 (*c* 0.10, MeOH); UV (MeOH) λ_{max} (log ε) 208 (3.67) nm; IR (ATR) ν_{max}: 2973, 2938, 2641, 1716, 1646, 1438, 1415, 1368, 1179, 1135, 1057, 1033, 997, 927, 815 cm^{-1}; ^{1}H and ^{13}C NMR data see Table 1; HRESITOFMS *m/z* 209.0784 $[M+Na]^+$ (calcd for $C_9H_{14}NaO_4$, 209.0784).

(*R*)-*O*-Isocrotonyl-3-hydroxyhexanoic acid (**2**): colorless amorphous solid; $[\alpha]^{24}_{D}$ -67 (*c* 0.10, MeOH); UV (MeOH) λ_{max} (log ε) 207 (4.15) nm; IR (ATR) ν_{max}: 2962, 2937, 1717, 1646, 1438, 1415, 1178, 1009, 970, 815 cm^{-1}; ^{1}H and ^{13}C NMR data see Table 1; HRESITOFMS *m/z* 223.0941 $[M+Na]^+$ (calcd for $C_{10}H_{16}NaO_4$, 223.0941).

(*R*)-*O*-(*Z*)-2-Hexenoyl-3-hydroxybutyric acid (**3**): colorless amorphous solid; $[\alpha]^{24}_{D}$ -38 (*c* 0.10, MeOH); UV (MeOH) λ_{max} (log ε) 209 (4.17) nm; IR (ATR) ν_{max}: 2962, 2934, 2874, 1717, 1640, 1456, 1414, 1178, 1059, 816 cm^{-1}; ^{1}H and ^{13}C NMR data see Table 2; HRESITOFMS *m/z* 223.0941 $[M+Na]^+$ (calcd for $C_{10}H_{16}NaO_4$, 223.0941).

(*R*)-*O*-Isocrotonyl-3-hydroxybutyric acid (**4**): colorless oil; $[\alpha]^{23}_{D}$ -43 (*c* 0.10, MeOH); UV (MeOH) λ_{max} (log ε) 208 (4.07) 274 (2.47) nm; IR (ATR) ν_{max}: 2985, 2937, 2643, 1710, 1644, 1435, 1414, 1291, 1175, 1057, 970, 815 cm^{-1}; ^{1}H and ^{13}C NMR data see Table 2; HRESITOFMS *m/z* 195.0627 $[M+Na]^+$ (calcd for $C_8H_{12}NaO_4$, 195.0628).

(*S*)-PGME amide of **4**, **4a**: ^{1}H NMR (500 MHz, $CDCl_3$) δ 7.34 (m, 5H, PGME-C_6H_5), 6.75 (d, *J*=6.9 Hz, 1H, PGME-N*H*), 6.31 (dq, *J*=11.5, 7.3 Hz, 1H, H3´), 5.70 (dq, *J*=11.5, 1.7 Hz, 1H, H2´), 5.57 (d, *J*=7.2 Hz, 1H, PGME-C*H*), 5.25 (brsex, *J*=6.3, 1H, H3), 3.72 (s, 3H, PGME-CH_3), 2.54 (dd, *J*=14.6, 7.0 Hz, 2H, H2), 2.09 (dd, *J*=7.2, 1.9 Hz, 3H, H4´), 1.34 (d, *J*=6.3

Hz, 3H, H4); HRESITOFMS *m/z* 342.1312 (calcd for $C_{17}H_{21}NNaO_5$ 342.1312).

(*S*)-PGME amide of **1**, **1a**: ^{1}H NMR (500 MHz, $CDCl_3$) δ 7.34 (m, 5H, PGME-C_6H_5), 6.75 (d, *J*=6.9 Hz, 1H, PGME-N*H*), 6.33 (dq, *J*=11.5, 7.3 Hz, 1H, H3´), 5.76 (dq, *J*=11.5, 1.8 Hz, 1H, H2´),5.56 (d, *J*=7.2 Hz, 1H, PGME-C*H*), 5.14 (brqui, *J*=6.3 Hz, 1H, H3), 3.72 (s, 3H, PGME-CH_3), 2.11 (dd, *J*=7.3, 1.8 Hz, 3H, H4´), 1.70 (m, 2H, H4), 0.92 (t, *J*=7.4Hz, 3H, H5); HRESITOFMS *m/z* 356.1468 $[M+Na]^+$ (calcd for $C_{18}H_{23}NNaO_5$, 356.1468).

(*R*)-PGME amide of **1**, **1b**: ^{1}H NMR (500 MHz, $CDCl_3$) δ 7.34 (m, 5H, PGME-C_6H_5), 6.75 (d, *J*=6.4 Hz, 1H, PGME-N*H*), 6.35 (dq, *J*=11.5, 7.3 Hz, 1H, H3´), 5.79 (dq, *J*=11.5, 1.8 Hz, 1H, H2´),5.56 (d, *J*=7.2 Hz, 1H, PGME-C*H*) , 5.17 (brqui, *J*=6.3 Hz, 1H, H3), 3.72 (s, 3H, PGME-CH_3), 2.11 (dd, *J*=7.3, 1.8 Hz, 3H, H4´), 1.67 (m, 2H, H4), 0.91 (t, *J*=7.4Hz, 3H, H5); HRESITOFMS *m/z* 356.1495 $[M+Na]^+$ (calcd for $C_{18}H_{23}NNaO_5$, 356.1468).

(*S*)-PGME amide of **2**, **2a**: ^{1}H NMR (500 MHz, $CDCl_3$) δ 7.34 (m, 5H, PGME-C_6H_5), 6.75 (d, *J*=6.4 Hz, 1H, PGME-N*H*), 6.34 (m, 1H, H3´), 5.75 (qd, *J*=11.5, 1.8 Hz, 1H, H2´), 5.57 (d, *J*=7.2 Hz, 1H, PGME-C*H*), 5.25 (m, 1H, H3), 3.72 (s, 3H, PGME-CH_3), 2.13 (dd, *J*=7.3, 1.7 Hz, 3H, H4´), 1.53 (m, 2H, H4), 1.49 (m, 2H, H5), 0.93 (m, 3H, H6); HRESITOFMS *m/z* 370.1625 $[M+Na]^+$ (calcd for $C_{19}H_{25}NNaO_5$, 370.1625).

(*R*)-PGME amide of **2**, **2b**: ^{1}H NMR (500 MHz, $CDCl_3$) δ 7.34 (m, 5H, PGME-C_6H_5), 6.75 (d, *J*=6.4 Hz, 1H, PGME-N*H*), 6.36 (m, 1H, H3´), 5.79 (dq, *J*=11.5, 1.8 Hz, 1H, H2´), 5.57 (d, *J*=7.2 Hz, 1H, PGME-C*H*), 5.28 (m, 1H, H3), 3.72 (s, 3H, PGME-CH_3), 2.16 (m, 3H, H4´), 1.49 (m, 2H, H4), 1.35 (m, 2H, H5), 0.91 (m, 3H, H6); HRESITOFMS *m/z* 370.1625 $[M+Na]^+$ (calcd for $C_{19}H_{25}NNaO_5$, 370.1625).

(S)-PGME amide of **3**, **3a**: ^{1}H NMR (500 MHz, $CDCl_3$) δ 7.34 (m, 5H, PGME-C_6H_5), 6.75 (d, J=6.4 Hz, 1H, PGME-NH), 6.24 (dt, J=11.5, 7.5 Hz, 1H, H3´), 5.72 (m, 1H, H2´), 5.57 (d, J=6.9 Hz, 1H, PGME-CH), 5.27 (brsex, J=6.3 Hz, 1H, H3), 3.72 (s, 3H, PGME-CH_3), 2.62 (dd, J=14.8, 5.6 Hz, 2H, H2), 2.61 (m, 2H, H4´) 1.73 (d, J=6.3 Hz, 3H, H4), 1.49 (sex, J=7.3 Hz, 2H, H5´), 0.95 (t, J=7.4 Hz, 3H, H6´); HRESITOFMS m/z 370.1625 $[M+Na]^+$ (calcd for $C_{19}H_{25}NNaO_5$, 370.1625).

(R)-PGME amide of **3**, **3b**: ^{1}H NMR (500 MHz, $CDCl_3$) δ 7.34 (m, 5H, PGME-C_6H_5), 6.75 (d, J=6.6 Hz, 1H, PGME-NH), 6.26 (dt, J=11.5, 7.5 Hz, 1H, H3´), 5.77 (dt, J=11.5, 1.6 Hz, 1H, H2´), 5.57 (d, J=7.1 Hz, 1H, PGME-CH), 5.30 (brsex, J=6.4 Hz, 1H, H3), 3.72 (s, 3H, PGME-CH_3), 2.63 (dd, J=14.7, 5.6 Hz, 2H, H2), 2.61 (m, 2H, H4´), 1.68 (d, J=6.3 Hz, 3H, H4), 1.49 (sex, J=7.2 Hz, 2H, H5´), 0.96 (t, J=7.4 Hz, 3H, H6´); HRESITOFMS m/z 370.1625 $[M+Na]^+$ (calcd for $C_{19}H_{25}NNaO_5$, 370.1625).

(R)-PGME amide of **4**, **4b**: ^{1}H NMR (500 MHz, $CDCl_3$) δ 7.34 (m, 5H, PGME-C_6H_5), 6.75 (d, J=6.9 Hz, 1H, PGME-NH), 6.33 (dq, J=11.5, 7.3 Hz, 1H, H3´), 5.76 (dq, J=11.5, 1.7 Hz, 1H, H2´), 5.57 (d, J=7.2 Hz, 1H, PGME-CH), 5.29 (brsex, J=6.3, 1H, H3), 3.72 (s, 3H, PGME-CH_3), 2.54 (dd, J=14.5, 6.7 Hz, 2H, H2), 2.09 (dd, J=7.2, 1.9 Hz, 3H, H4´), 1.32 (d, J=6.3 Hz, 3H, H4); HRESITOFMS m/z 342.1312 (calcd for $C_{17}H_{21}NNaO_5$ 342.1312).
